# Consequential insights for advancing informal STEM learning and outcomes for students from historically marginalized communities

**DOI:** 10.1057/s41599-024-02797-w

**Published:** 2024-03-02

**Authors:** Claudia McLaughlin Ludwig, Rebecca A. Howsmon, Shelley Stromholt, Jacob J. Valenzuela, Rachel Calder, Nitin S. Baliga

**Affiliations:** 1 Institute for Systems Biology, Seattle, WA, USA.; 2 Ballard High School, Seattle Public Schools, Seattle, WA, USA.; 3 Aspect Research + Evaluation, Seattle, WA, USA.; 4 OMNY Health, Atlanta, GA, USA.; 5 Departments of Biology and Microbiology, University of Washington, Seattle, WA, USA.; 6 Molecular and Cellular Biology Program, University of Washington, Seattle, WA, USA.; 7 Lawrence Berkeley National Lab, Berkeley, CA, USA.

## Abstract

Consequential STEM experiences in informal settings can address issues of equity by fully engaging historically marginalized high school students in complex socio-scientific issues. However, inclusive and effective programs are in high demand, and there is little research on what specific aspects, context, and timeframes are most important when scaling these experiences. Using a mixed method approach, this study demonstrates that students make significant gains, in the short and long term, through in-person and remote informal programs ranging between 22-h and 320-h. Progress across STEM learning constructs is attributed to authentic research experiences, students’ connections to STEM professionals, direct hands-on participation in projects, and group work. Relative to formal education settings, research-based informal STEM programs can be implemented with minimal resources, can maintain effectiveness while scaling, and work towards addressing the societal challenge of improving STEM learning and outcomes for high school students from historically marginalized communities.

## Introduction

As a global community facing the challenges of climate change, healthcare, and other complex socio-scientific issues, it is imperative that all members of society have opportunities to develop STEM literacy so they can be well-equipped to make informed decisions and take action. STEM (science, technology, engineering, and mathematics) literacy, “the dynamic process and ability to apply, question, collaborate, appreciate, engage, persist, and understand the utility of STEM concepts and skills”, is a life-long process that is influenced by learning opportunities in both formal (in-school) and informal (out-of-school) settings (Jackson et al. 2021; Mohr-Schroeder et al. 2020). Consequential experiences with STEM, including engagement in scientific thinking, practices, and collaborations, prepare students to learn, live, work, and engage in an increasingly STEM-centric society (National Institutes of Health, 2019). Therefore, there is an overwhelming consensus that opportunities to develop STEM literacy are critical for all students, regardless of their future roles in the modern workforce (Feinstein et al. 2013; National Research Council, 2012).

Unfortunately, equity in STEM education remains a challenge. The stereotypes of who does and does not belong in STEM (Jackson et al. 2021) contribute to persistent disparities in STEM education access and engagement for underrepresented groups (Eagan et al. 2014). The opportunity gap for women, Black, Latinx/a/o, and Indigenous peoples, as well as their historical marginalization in science-related higher education and careers, is well documented (Building Engineering and Science Talent, 2004; National Institutes of Health, 2019; National Research Council, 2011, 2012; National Science and Technology Council, 2021; National Science Board, 2018; Washington State STEM Education Innovation Alliance, 2019). While progress has been made since 1993, as shown by the National Science Board, the representation of each of these communities in science and engineering fields is not proportional to their share of the U.S. population (Fry et al. 2021; National Center for Science and Engineering Statistics, 2021; National Institutes of Health, 2019; National Science Board, 2018; Valantine et al. 2016). The implications of these inequities are far-reaching, impacting who is directly involved in scientific research as well as who benefits from the research findings (Kearney et al. 2021). For this paper, we use the term “historically marginalized communities” (HMC) in reference to individuals who identify as female, Black, Latinx/a/o, members of Indigenous communities, individuals with dis(abilities), and/or those affected by poverty who also experience discrimination and exclusion from STEM opportunities (National Institutes of Health, 2019).

To address these issues of inequity, Systems Education Experiences (SEE) within the nonprofit scientific research organization, Institute for Systems Biology (ISB), has provided rigorous, non-episodic STEM learning experiences for high school students since 2003. These experiences were initially developed as 320-h interdisciplinary, hands-on summer internships. As student interests shifted and the number of applicants to these longer-duration summer internships continued to grow, the program grew in response by designing and introducing four additional STEM experience models over 5 years. All new STEM experience models were co-created with students and teachers using the same program design principles and overall goals, but the content, timing, and context (in-person versus online) were varied. Ambassadorships began in 2016, 90-h courses in 2019, 40+ h workgroups in 2020, and 22-h short courses in 2021. Two noteworthy implications of these alternate STEM experience models were that (i) they scaled up high school student participation by 20-fold (Day et al. 2021; C. Ludwig et al. 2020; Systems Education Experiences 2023b; Tomky, 2016), and (ii) despite their unique content, they all advanced students’ STEM interest, proficiency, and 21^st^ Century Learning Skills, while broadening participation in addressing complex, relevant, and contemporary problems.

The expansion of SEE to include four STEM experience models provided an opportunity to study whether the design and implementation of each model were equitably supporting youth, especially those from HMC, in developing STEM literacy and sustaining interest in STEM. Here, we report on the analysis of pre and post experience data collected from 239 high school students who participated in one of four STEM experience models (herein called “STEM experiences”) provided by SEE between 2003 and 2021. The data served to address three key study questions:

1. What *progress* on STEM pathways do students from HMC make as a result of participation in informal STEM learning experiences?
2. What *successes and challenges emerge* when young people from HMC engage in authentic STEM experiences?
3. What *aspects* of informal STEM learning experiences support young people from HMC in making progress on STEM pathways?

The findings from this study meet an important need within the STEM education community by providing researchers and informal science educators consequential and practical insights for supporting equitable STEM learning that can be integrated into new and existing STEM experiences. These consequential insights will help ensure all members of our society, especially those from HMC, are prepared to advocate for the needs of individuals and communities, and have the knowledge and skills to act on these needs by designing creative and innovative solutions to the complex global issues we face now and in the future.

## Theoretical framework with embedded literature review

The last decades of STEM education research have provided sound theoretical guidance toward designing and implementing equitable STEM learning settings. The cross-disciplinary research team drew from numerous studies and theories to provide a foundation for this research and specifically to inform data collection and analysis. The team began with an examination of historical qualitative and quantitative SEE data and by analyzing national trends from the National Science Board 2018 Science and Engineering Indicators report (National Science Board, 2018). The team also looked at sociocultural theories and agreed to define learning as the transformation of participation within ongoing activity in communities of practice (Bloch et al. 1994; Lave, 2012; Rogoff, 2003). Current research has indicated that learning settings are typically socially and materially constructed in ways that privilege specific power and knowledge structures, often those associated with white, middle-class discourses and values (Barton et al. 2008; Brandt, 2008; Moje et al. 2001). To address this issue, the program design principles were reevaluated while considering current literature on programs that aim to broaden STEM participation in consequential ways, provide equitable and authentic opportunities “to access the existing STEM knowledge base, contribute to the generation of STEM knowledge, and/or realize the benefits of STEM” (C. Garibay and Teasdale, 2019). This study was informed by others’ research demonstrating that broadening access through consequential participation can provide opportunities for young people to:

- Engage in authentic STEM-based practices that crosscut between disciplines historically unavailable to them, as in developing models or using computational and systems thinking (National Research Council, 2012),
- Explicitly contest historical narratives of who can do science and develop science-linked identities (H. Carlone, 2017; Rahm and Moore, 2016; Thompson, 2014), and
- Make direct connections to their everyday lives and better understand ways they can use science to advocate for their communities (Stromholt and Bell, 2018).

Additionally, this study draws on Schreiner’s (2010) concept of thriving; the *Equity-Oriented Conceptual Framework for K-12 STEM Literacy* (Jackson et al. 2021); and research showing that consequential STEM learning can be achieved through designed experiences in both formal classrooms and in informal settings such as museums, science centers, and research institutes. These frameworks are described more thoroughly below in the study constructs.

Informal settings, defined as places where voluntary participation in learning happens during out-of-school time, are an important part of the STEM learning ecosystem (Hofstein and Rosenfeld, 1996; National Research Council, 2015). While there are numerous programs across the nation that provide these types of learning experiences, there remains a need within the informal STEM education community to understand *what aspects* of these learner engagements support youth, especially those from HMC, in developing STEM literacy and sustaining interest and participation in STEM. Based on this need, these theoretical frameworks, and the research-based program design principles, the team selected six overlapping STEM equity *constructs* as the focal point of this study in order to answer the three research questions. Table 1 and the sections below further describe these overlapping constructs and include a literature review highlighting related studies and the rationale for inclusion in this study.

### Engagement.

Engagement has been broadly described as “the behaviour toward, relationship with, and a commitment to learning” (Solomonides and Reid, 2009). In both informal and formal settings, engagement is then related to identity, motivation, authentic tasks, relevance, and interest, among other constructs that contribute to our understanding of how and why learners participate in activities - or why they do not (James Bell et al. 2019). Students’ engagement was investigated as a means to determine their level of interest, satisfaction and commitment to learn and make progress towards program goals.

### Awareness and intent (two key constructs within STEM pathway progression).

A consensus report from the National Academies of Sciences, Engineering, and Medicine concluded that “success is achieved when all students who are interested in STEM: are able to make informed decisions about the best course of study for them based on interests, motivation, and career aspirations; understand the variety of potential career pathways that come with STEM degrees; have a clear understanding of STEM content and practices; do not face unreasonable barriers along their pathways that discourage them or make progress impossible; and are aware of connections between STEM and societal issues and concerns” (National Academies of Sciences, Engineering, and Medicine, 2016). Thus, intent to pursue a STEM career is a key factor in understanding a student’s probable pathway. As such, connecting “intent” to other constructs such as awareness, interest, identity and social capital, is an active area of research (Christensen and Knezek, 2017; Lin et al. 2018). To investigate student success in pursuing a STEM pathway, we integrated data collection tools that consider students’ awareness of and intent to pursue a STEM pathway. In some cases, these are analyzed together and in others separately based on the context and research question.

STEM pathways generally refer to the movement of students along “multiple pathways” toward STEM degrees (National Academies of Sciences, Engineering, and Medicine, 2016). Though studies of STEM pathways focus on higher education, young people’s preparation for and decisions to embark on STEM pathways begin much earlier (Maltese and Tai, 2011; Sadler et al. 2012; Tai et al. 2006). All young people are part of a STEM ecosystem that includes experiences in and out of school that influence their intent to pursue and persist in STEM degree programs. However, previous work has shown that disparities in access and engagement exist for HMC beginning in K-12 and continuing into higher education (Ashford et al. 2016; Eagan et al. 2014; Sadler et al. 2012). In recognition of these inequities, a consensus report from the National Academies of Sciences, Engineering, and Medicine calls issue to the historical focus on graduation rates as the defining factor in STEM success, as they do not take into account the institutional context and student characteristics (National Academies of Sciences, Engineering, and Medicine, 2016). The authors instead draw heavily on the concept of *thriving* as a desirable goal in STEM pathways, a concept that attends to dimensions such as how students are academically engaged, make efforts toward goals, and connect to their community (Schreiner, 2010). In this study, we combined the studies discussed above to provide a strong foundation for STEM pathways research to understand how learners in the SEE program are supported, or not, by their informal STEM learning experience to make progress toward STEM success.

### Identity.

Identity is constructed and reinforced by the social processes and situated contexts in which learners participate (Nasir and Hand, 2006; Wortham, 2004). STEM identity is, therefore, dynamic and actively negotiated in different places and contexts (Jamie Bell et al. 2019; Kim et al. 2018; Seyranian et al. 2018). Interest and engagement with STEM and STEM-related careers can then be linked in part to current and future identities or how young people consider themselves in relation to narratives of “what it means to be a science person” (Kang et al. 2019). In any disciplinary setting, learners must negotiate the tension between their everyday ways of being with ways of being in science communities (H. B. Carlone and Johnson, 2007; Holland et al. 1999). This includes deciding what kind of science-oriented person they want to be and engaging in the appropriate practices to move toward that goal. Because of the social nature of this negotiation, learner identities are context-dependent and affected by the goals, assumptions, and recognition of others (Valdez et al. 2020). Students’ perceptions of their identities were investigated as a key piece of predicting whether or not they made progress towards being able to consistently and fully participate in STEM contexts.

### Social capital.

Social capital is the set of intangible resources powered by a person’s interpersonal relationships or social institutions that lead to productive advances that would otherwise be unlikely (Coleman, 1988). Two-way, or multi-directional, trusting, “developmental relationships” are particularly important for helping youth build social capital. These relationships provide opportunities for building close connections that allow young people to discover who they are, cultivate abilities to shape their own lives, and learn how to engage with and contribute to the world around them (Search Institute, 2020). Social capital is likely impacted for students from HMC with access to fewer social resources such as mentors and peers with shared cultural identities or experiences (Hawes, 2011).

### 21st-century learning skills.

21st Century Learning Skills and the disciplinary practices of science, including interactions, tools, and language, have some common ground (Duschl et al. 2007). 21st Century Skills are literacies and skills like critical thinking, problem-solving, communication, and collaboration that are considered necessary for success in projected future workforces (Partnership for 21st Century Learning, 2020; Rich, 2010). This study attends to learners’ opportunities to practice disciplinary-specific 21st Century Skills that support them in collaboratively addressing relevant, complex problems. Based on the described collective insight from previous research, in this study, we measured and analyzed changes in these six constructs through retrospective pre-post surveys and interviews.

## Methods

### Participants.

**Fig. 1:**
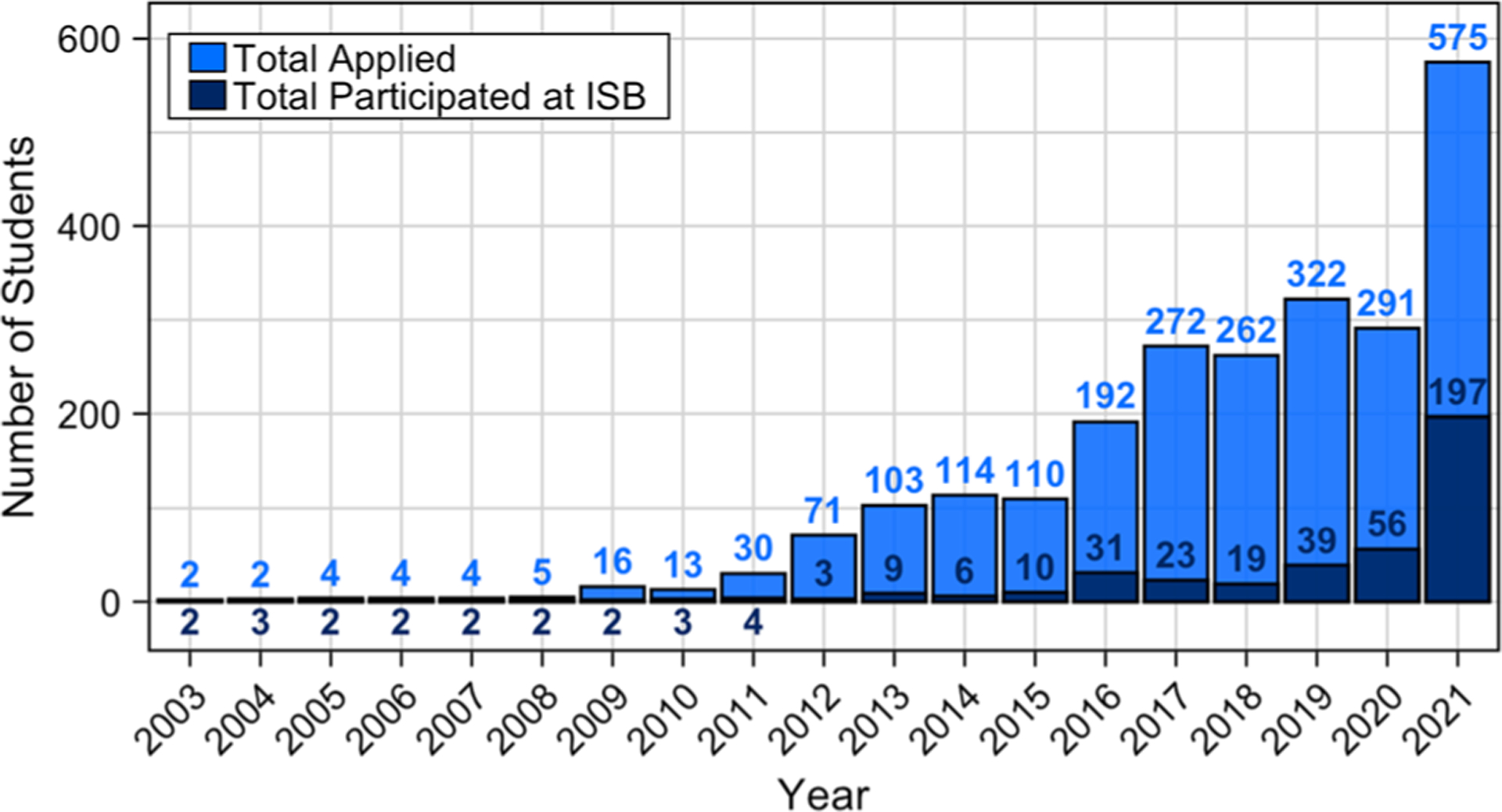
Number of SEE student applicants and participants per program year. Bars and numbers represent the total number of high school student applicants and participants in summer experiences per year. A total of 415 students, who participated 2003–2021 are the focal point of this study.

SEE has provided immersive experiences for over 800 high school students since its inception in 2003 (Systems Education Experiences, 2023a). Of these students, 415 completed immersive experiences directly at ISB and were the focal point of this study (Fig. 1 with participant numbers in dark blue). 80% of these 415 students were from HMC. This study includes data from 239 student participants (58% of 2003–2021 participants). Data was collected from 56 alumni who completed an experience with SEE between 2003–2019 (internship or ambassadorship). Additionally, data was collected from 183 students who directly engaged in one of four STEM experience models between 2019–2021: 320-h internship, 90-h course, 40+ hour workgroup, or 22-h short-courses. All experiences were designed to support high school students in learning about the principles and practices of the systems science research at ISB, while attending to the varied needs, interests, and availability of high school students. Each experience included opportunities for students to learn about systems, develop professional skills, explore new topics, collaborate on team projects, and network socially and professionally, regardless of duration and content focus. Table 2a, b provides an overview of each experience, and more detailed descriptions can be found in the Supplemental Material.

### Participant application and hiring process.

For all experiences, the application process was the same. Students submitted their application online, which included responding to a series of question prompts, selecting the experience(s) they were interested in, uploading a copy of their resume and unofficial high school transcript, and having a teacher or supervisor submit a letter of recommendation on their behalf. Completed applications were reviewed by SEE staff, and a subset of applicants were invited to participate in phone and/or in-person interviews. Final selections for all experiences were made by staff and scientists based on an established rubric that took into consideration information from each touchpoint, including the students’ family background, social environment, previous STEM experience, etc. Final decisions were communicated to all applicants in May, and experiences ran throughout the out-of-school summer months of June, July, and August. For all experiences, priority acceptance was given to students from HMC and/or students who had limited previous STEM experience.

### Data collection and analysis.

For this study, two online surveys (SurveyMonkey) were drawn from existing and validated tools that align with the theories and constructs described above. Statistics were performed using a two-sided *t* test to evaluate significant differences. One survey was used with program alumni, and the other for 2019–2021 participants. Interviews were also completed for 2019–2021 participants.

#### Alumni survey.

The retrospective alumni survey consisted of a series of items to understand the impact of the SEE experiences on the STEM pathways of alumni in comparison with their experiences in high school and other extracurricular STEM activities. The survey was emailed to students who had previously participated in a SEE experience between 2003 and 2019. Respondents were asked to rate the extent to which their experience supported their progress toward STEM success. The components of the STEM pathways framework were used as prompts, and students rated each using a scale of 1–4 (Not at all, A little, Somewhat, A lot). To make comparisons, descriptive statistics were used to generate means for each thriving component in relation to each experience. Not all respondents responded to each item. For this analysis, statistics are calculated for each item by the number of respondents who completed that item. Measures were taken to mitigate the fact that student respondents may have felt compelled to give answers that portray SEE in a positive light, as they were asked to respond to a survey about their experience that was sent out by SEE staff. These measures included both the introduction and framing of the survey and its purpose, and in the order and phrasing of the survey items. Despite these efforts, we acknowledge the data presented will be somewhat skewed, and thus, t-tests were performed to compare the three experiences to the best of our abilities.

#### Participant surveys.

To capture student participants’ before and after attitudes towards STEM and their futures, we adapted a variety of validated tools and compiled them into a retrospective pre-post survey (Gouldthorpe and Israel, 2013; Hoogstraten, 1982; Klatt and Taylor-Powell, 2005; Little et al. 2020) that asked students to use a 4-point Likert scale to first reflect on how they felt after completing their experience (post), then reflecting retrospectively on how they felt before they began the experience (pre). The number of items for each construct ranged from 2 to 7, with some constructs addressed by both the pre-post items as well as the post-only items (Table 3). At the completion of each experience in 2019, 2020, and 2021, all 262 participating high school students were provided a link to the online survey via email or during the final minutes of the experience. Completion of the survey was optional, responding to each item was not required, and all responses were anonymous. As such, the sample size (*n*) for the reported results differs for each experience and construct.

To identify themes in student responses or gaps in the data with particular relevance to the STEM pathways framework, the 2020 and 2021 surveys also asked students to respond to a set of open-response questions aimed at identifying which aspects of the programs contributed to their Likert scale ratings. These questions were developed for a focus group protocol in 2019 and adapted for surveys as part of the shift to online programming in 2020.

To ensure validity of the data collection tools and the reliability of the results, we used or adapted existing instruments for the outcomes of interest. Our focus on equity, as well as on contemporary science, dictated that, to some extent data collection tools and items needed to be developed specifically for this study. In those cases, steps were taken to ensure ecological validity, ensuring as much as possible that the tools accurately reflected the settings and experiences under examination. For example, by carefully tying survey items to the specific program strands students were experiencing, at the time they were experiencing them, we aimed to create and revise tools that accurately predicted youth engagement in the programs.

The 22-h short courses consisted of two connected experiences, Tier 1 (2 h) and Tier 2 (20 h). The time between students’ Tier 1 and Tier 2 experience varied between 2 and 6 months depending on their chosen Tier 1 workshop date and Tier 2 course. Students completed a retrospective pre-post survey after completing Tier 1 (data not shown) as well as after Tier 2. Due to purposeful anonymization, we did not track student survey responses longitudinally (i.e., connecting their Tier 1 and Tier 2 survey responses). Therefore, we chose to capture three timeframes with the Tier 2 survey: before Tier 1, before Tier 2, and after Tier 2. For this publication, we chose to showcase the change in attitude using the two endpoints (before Tier 1 = pre, after Tier 2 = post) and exclude the midpoint data to align with the data from the other programs. In this way, all pre data represents students’ attitudes before starting their experience, and all post data represents students’ attitudes after completing their experience.

Mean values across all student responses within an experience were calculated for each construct’s pre and post responses; mean values were then used to calculate percent change. Descriptive statistics were calculated for all Likert scale items, including means. To identify emergent themes from the qualitative responses, the study team coded a subset of responses to develop an initial coding scheme, then revised these codes through discussion and triangulation with the full data sets. Student respondents were also asked to select the race/ethnicity and gender they identify with and/or to describe their race/ethnicity and gender using an open-response option. The four-point Likert survey responses for all students were replaced with their ordinal value (Strongly Agree = 4, Agree = 3, Disagree = 2, Strongly Disagree = 1). Individual pre and post values for each student were calculated by averaging the ordinal values across all items within each construct. Percent change was calculated for each construct by comparing the mean values of the pre vs post survey results for each program. To accurately compare pre and post responses, individual student data were removed across all items within a construct if a response to both the pre and post versions of an item were not provided. As a result, the number of students (*n*) included in the analysis for each construct differs.

## Results

### Survey results—alumni.

The majority of alumni progressed along STEM pathways and attributed their success to SEE features such as the authentic, hands-on experiences and direct connection to scientists. To assess the specific progress on STEM pathways that could be attributed to their participation, we asked 150 student alumni from each SEE experience from 2003–2019 to complete an online survey. Fifty-six of the 150 student alumni (37%) responded to the survey, which included 40 women and 11 men (5 did not respond to the gender identity question); 62% identified as being from a HMC.

In the first portion of the survey, respondents were asked to provide details on their academic and career path. Fifty-four of the 56 respondents (96%) reported having taken an advanced STEM course in high school; 98% went directly from high school to their undergraduate degree; 42 (78%) stated majoring or minoring in a STEM subject area for undergraduate, with biological sciences as the most common degree reported. Forty-seven (84%) stated they were currently or were planning to pursue a medical, graduate, or professional degree, with ‘Science or research’ (58%) and ‘Healthcare’ (38%) being the primary fields of work identified (even if in-school or unemployed).

**Fig. 2:**
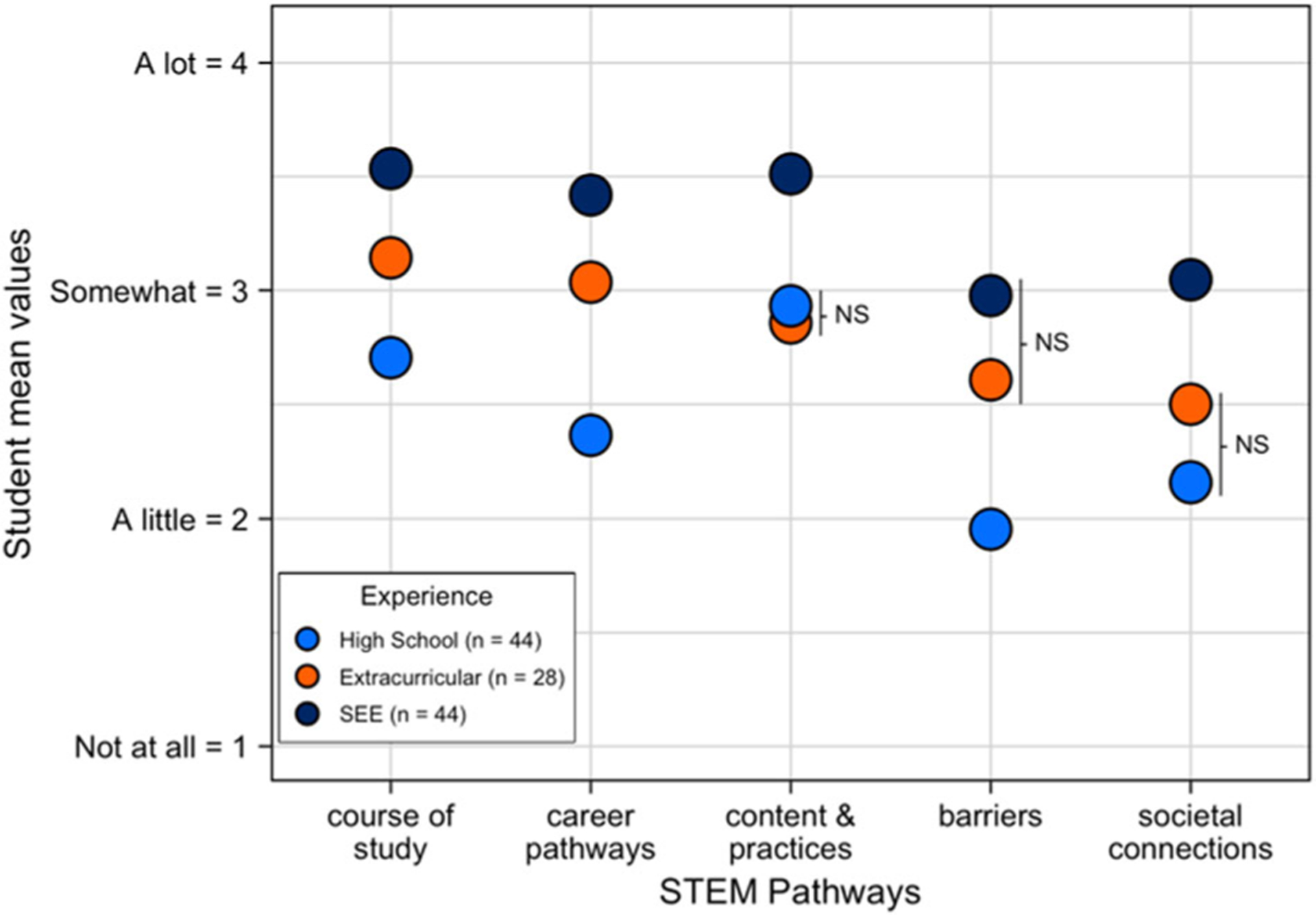
SEE provides unique opportunities for students to thrive in STEM pathways. Mean values were calculated for each experience type (SEE, Extracurricular, High School) in terms of how each contributed to the five components of the thriving framework (*x* axis). Student alumni responded anonymously to each question using a 4-point Likert scale (4 = A lot, 3 = Somewhat, 2 = A little, 1 = Not at all). All comparisons are statistically significant, *p* ≤ 0.05, based on *t* tests, except those noted as not significant (NS).

In the second portion of the survey, respondents were asked to rate the extent to which their experience with SEE, their high school experience generally, and another extracurricular STEM experience, if they had one, helped them to make progress on the following STEM Pathway components: (1) make informed decisions about their course of study; (2) understand potential STEM career pathways; (3) have a clear understanding of STEM content and practices; (4) understand potential barriers in STEM and how to address them; (5) become more aware of connections between STEM and societal issues and concerns. Using a 4-point Likert scale, SEE alumni rated their experience with ISB as statistically different from their high school experiences in all five STEM pathway components (Fig. 2), with the largest differences in how they learned about “career pathways” (mean = 3.4 vs 2.4) and the “barriers” they might experience (mean = 3.0 vs 2.0). Those who had other extra-curricular STEM experiences (*n* = 28) rated their experience with SEE as statistically different in four of the five components, particularly in how they learned “content and practices” (mean = 3.5 vs 2.9), as well as how they learned about the “societal connections” to STEM (mean = 3.0 vs 2.5). The two components with the lowest ratings across all experiences were “barriers” and “societal connections.” When the data was disaggregated by gender or race/ethnicity we found experiences with SEE consistently remained statistically different from high school experiences across all thriving components. Experiences with SEE also remained statistically different from other extracurricular experiences in supporting students’ “understanding of STEM content and practices.”

### Aspects of SEE alumni experiences that contributed to STEM pathway progression.

To understand what aspects of SEE contributed to alumni students’ STEM pathway success, the second portion of the retrospective alumni survey prompted students to identify specific aspects of their experience that influenced how they rated each of the components of the framework and how that aspect impacted their STEM pathway success. A representative selection of student responses is provided in Table 4. Based on the information provided in their survey responses, 80% or more of these students are from HMC, even though only 62% are directly identified as being from an HMC on the associated survey prompt. In general, student alumni shared that their course of study, understanding of potential career pathways, barriers, and societal connections were all informed by their direct and indirect interactions with scientists, whose backgrounds spanned a variety of STEM disciplines. They attributed these interactions to: (i) helping identify and trigger interest in majors and career paths that were previously unknown to them, (ii) “illuminat[ing] the different barriers and career options in the STEM field”, and (iii) “see[ing] how the science [they did] could possibly impact the world to a great extent.” Alumni also shared that SEE provided them with opportunities to gain experience in an array of authentic STEM content and practices, including “how to pipet, use a centrifuge, manipulate data in Excel, keep a lab notebook” and “proper lab etiquette.” They also acknowledged the importance of “having the experience of working” and “observ[ing] a lot of analytical/critical audiences responding to preliminary data.”

### Survey results - four STEM experience models.

Of the 262 students who participated in one of the four experiences, 183 (70%) completed all or part of the survey; 153 responded to the question on gender identity, with 74% identifying as female and 1% identifying as non-binary; and 99 responded to the question on race/ethnicity (see Supplementary Tables 1 and 2). Results were aggregated based on the STEM equity constructs of engagement, awareness, identity, intent, social capital, and 21st Century Learning Skills, then analyzed to identify successes and challenges that emerged. The data was intentionally left demographically aggregated as the number of students in some experiences was small enough that disaggregating the data by gender, race, or ethnicity could potentially result in data being identifiable.

#### Engagement.

**Fig. 3:**
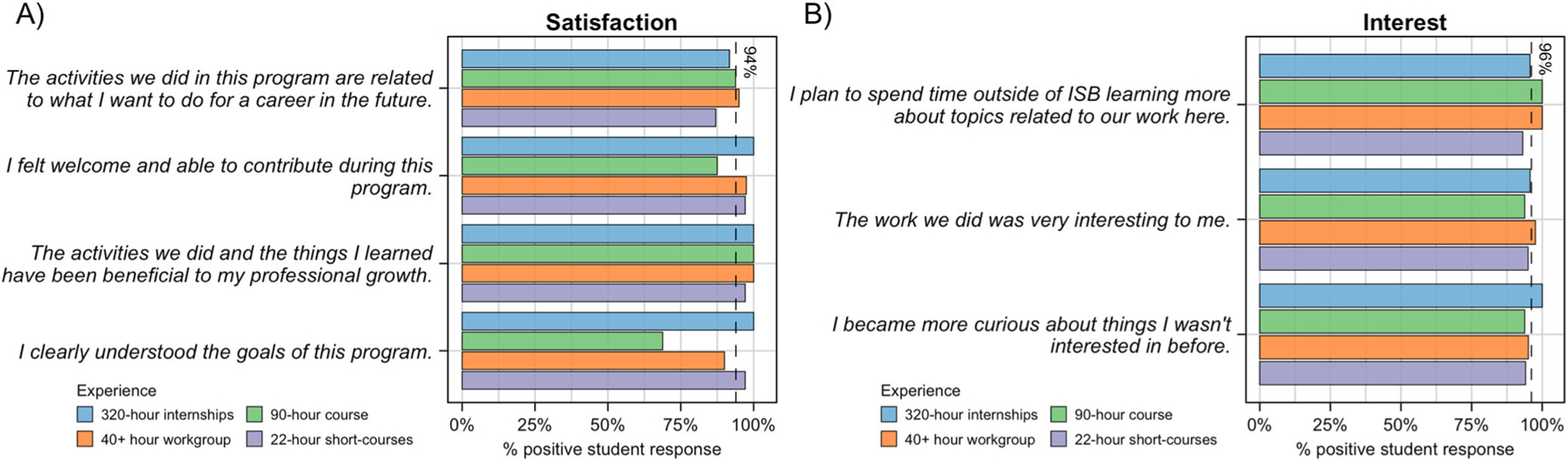
Post-only student survey results for questions related to engagement. Students responded to four post-only survey questions that serve to assess satisfaction **A** and three to assess interest **B** upon completion of participation with one of four SEE experiences. Presented are the percentage of positive responses (“Strongly Agree” or “Agree”) for each question, color-coded by experience. Also presented is a dashed line showing the averages, which are 94% positive for satisfaction and 96% positive for interest across all experiences.

As demonstrated in Fig. 3, students participating in an experience with SEE were satisfied (Fig. 3A) and interested (Fig. 3B) (broadly referred to as engagement), with an average of 94% and 96% positive for satisfaction and interest across all experiences (dashed lines), respectively. Students participating in the 90-h course overall rated the item “I clearly understood the goals of this program” lower than all other items related to engagement (69% positive; Fig. 3A). Deeper analysis of the data reveals that of the five students who did not respond positively to this item, four responded positively to all other items related to satisfaction and interest, and all five demonstrated gains across all other constructs.

#### Awareness.

**Fig. 4:**
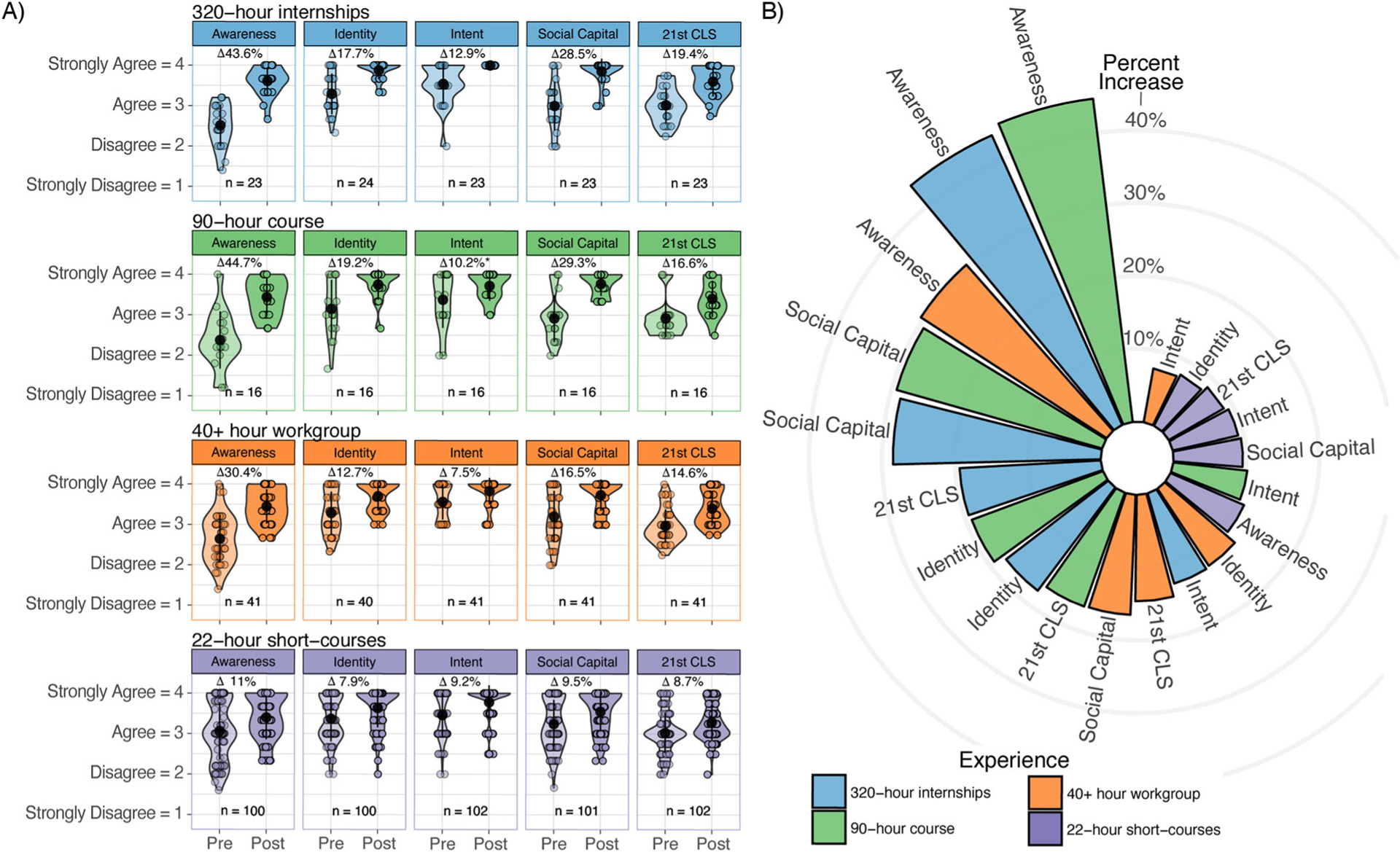
Pre and post experience student survey results within each construct aligned with STEM success. **A** Scatter plots with individual circles representing the average of an individual student’s responses to questions related to each of the five constructs before their experience (pre) and after their experience (post). Violin plots overlay the scatter plots and are color-coded with lighter coloring for pre data and darker coloring for post data. Students responded anonymously to each question using a 4-point Likert scale as shown on the left. Solid circles represent pre and post means. Percent change is presented for each construct; all values (except as noted by *) are statistically significant, ≤0.001 based on the student’s paired *t* test. *Statistical significance is 0.04. Mean pre and post values are provided in Supplementary Table 3. **B** Circular bar chart summarizes the percent change for each construct. Supplementary Fig. 1 shares a line chart as an alternative view, from the circular bar chart, summarizing the percent change for each construct.

Percent change calculations demonstrate students in all four experiences increased their awareness of STEM careers and pathways during their experience with SEE (Fig. 4). While longer-duration experiences (320-h, 90-h and 40+ hour) had the greatest impact on students’ awareness, it is important to note that the lower percent change for the 22-h short courses (11%) is the result of a higher pre-value mean for this experience: 3.1 (22-h) compared to 2.5 (320-h), 2.4 (90-h), and 2.6 (40+ hour), as the post-value means for all experiences are similar (3.4, 3.6, 3.4, 3.4 respectively) (Supplementary Table 3).

#### Identity.

Post-only survey responses demonstrate the majority of students (≥83%) found their experience conducive to building a positive STEM identity, including that the work they did was relevant, helped them see themselves working in a STEM career, and that they identified with the professionals they interacted with (Fig. 6). Consequently, students’ pre-post data (Figs. 4 and 7) demonstrates an increase in their STEM identity as a result of their experience with SEE. Specifically, overall percent change values for each experience are 17.7%, 19.2%, 12.7%, 7.9%, respectively. These percentages are relatively small due to the majority of students entering into their experience with an established STEM identity (mean pre-values for each experience are 3.3 (320-h), 3.2 (90-h), 3.3 (40+ hour), 3.4 (22-h)—Supplementary Table 3). Thus, even though the values did increase at the close of the program (mean post values for each experience are 3.9 (320-h), 3.8 (90-h), 3.7 (40+ hour), 3.6 (22-h) —Supplementary Table 3), the change is less dramatic.

#### Intent.

As with identity, students from all experiences reported a high degree of interest and confidence, broadly referred to as intent, in pursuing a STEM career before their experience (mean pre values are 3.5 (320-h), 3.4 (90-h), 3.6 (40+ hour), 3.5 (22-h)—Supplementary Table 3). As such, the percent change for intent is also relatively small (Fig. 4). All students in the 320-h and 40+ hour experiences responded positively to the prompt “I would consider a career in STEM” in their pre-assessment (Fig. 8). In contrast pre values for this prompt were 94% and 98% for the less selective 90-h course and 22-h short-courses, respectively. However, post values across all experiences were nearly 100%.

#### Social capital.

Aggregated data across all three items related to social capital demonstrates a positive percent change across all experiences (Fig. 4 and Table 5). This positive trajectory is the result of significant gains across two of the three pre-post items: “I have talked to an engineer, scientist, or someone who works in technology or math about their job” and “I know someone outside of school who can help me learn more about STEM” (Fig. 9). Little change was seen in response to the prompt “My family and/or friends encourage me to think about a career in STEM” due to a high percent positive in both pre and post responses. Interestingly, across all experiences, only six students stated “Disagree” to this question in their pre response, with four shifting their response to “Agree” (*n* = 3) or “Strongly Agree” (*n* = 1) in their post survey. The other two maintained their “Disagree” response for pre and post, however, they both stated they plan to stay connected to “one or more of [their] mentors” as well as “peers in [their] cohort” (Table 5).

#### 21st century learning skills.

Overall, students reported a strong foundation of professional skills upon entering their experience that was further developed during their experience with SEE (Fig. 4). Analysis of pre-post changes for each question demonstrates students developed confidence to “make changes when things do not go as planned” and to “manage [their] time wisely when working on [their] own” (Fig. 10). Students also reported high levels of confidence in their ability to “work well with different types of people” both before and after their experience. The most variable results were in response to the prompt “I take risks and try new things.” Education research demonstrates that risk-taking can buffer the negative effects of stereotype threat and is an important part of learning (Petzel and Casad, 2020; Shahin et al. 2021). During programming, SEE staff noticed students taking risks and responding to uncertainty and ambiguity. Staff consciously designed a safe and supportive environment while pushing students to take risks and think creatively during project work. The data in Fig. 10 demonstrates students across all experiences made improvements in 21st Century Learning Skills. However, the lower post results with regard to taking risks (88% in the 90-h course and 89% in the 22-h course) suggest students felt slightly less confident in this skill than others.

#### Remote vs In-person experiences.

During the course of this study, we shifted to remote experiences due to the COVID-19 pandemic and used this shift to study whether it impacted student outcomes. Of the four experiences, only the 320-h internship data is made up of both in-person and remote cohorts as the aggregated data comprises three student cohorts over 3 years; 2019 was fully in-person whereas 2020 & 2021 were fully remote. Despite the different modes, the overall experiences were quite similar in size of cohort (*n* = 10 (2019), 8 (2020), 6 (2021)), length of time, and overall design. To explore whether the mode of experience influenced student impacts, we disaggregated the data into individual cohorts (Table 6). Percent change between pre and post responses within each internship cohort was significant (*p* ≤ 0.05, paired *t* test). Starting point (pre values) comparison across cohorts demonstrates some significant difference in Identity and 21st Century Learning Skills between the 2019 and 2021 cohorts, representing the unique background experiences of students within each cohort. More importantly, cross comparison of outcomes (post values) across cohorts demonstrates little difference, with the exception of 21st Century Learning Skills in which the 2021 cohort reported significantly lower values than the 2019 and 2020 cohorts.

To better understand the source of these differences, we looked at percent positive for each of the four questions related to 21st Century Learning Skills (Fig. 11). These results demonstrate that 100% of students from all three cohorts developed confidence “to make changes when things do not go as planned” as a result of their experience, and sustained or improved their ability to “manage [their] time wisely when working on [their] own” and to “work well with different types of people.” It is important to note that because of the small size of the 2021 cohort (not all students answered all questions: *n* = 6), the lower percent positive for the item “I work well with different types of people” is the result of a single student who responded “Disagree” to this question. This student responded positively to all other post items, and their “Disagree” response is actually an increase from their pre response of “Strongly Disagree”, thus this response still represents a positive impact on the overall learning for that particular student. Similarly, 100% of the 2019 and 2021 cohort students felt positive they could “take risks and try new things” while only 88% of the 2020 cohort students felt the same. Again, because of the small cohort size (*n* = 8), this seemingly significant impact is the result of a single “Disagree.” In this particular case, this post response is a sustained feeling from their pre response which was also “Disagree”, signifying this is a skill the student is still developing.

### Aspects of the experiences that contributed to students’ development of STEM literacy and interest in STEM.

To understand what aspects of the different experiences contributed to their STEM learning, the second portion of the retrospective pre-post participant survey prompted students to identify the specific aspects of their experience that impacted their growth in the aforementioned constructs, including “Develop identity as someone who can do STEM” (Identity), “Becomes more interested in STEM or STEM careers” (Intent), “Learn about career options and how to get there” (Awareness), “Develop social capital” (Social Capital), and “Learn 21st century skills” (21st Century Learning Skills). All responses were coded to identify activity themes, and then the percentage of students who attributed each theme to a supported construct was calculated based on the total number of respondents for each respective construct (Table 7).

As demonstrated in Table 7, the constructs Identity and Intent were supported by every activity theme identified by student respondents. The two activity themes that were identified by students as having the greatest impact on their identity and intent were “Using scientific tools and materials” (26%) and “Guest speakers, interviews, and career-connected videos” (29%). Interestingly, “Guest speakers, interviews, and career-connected videos” was also identified as supporting students in all other assessed constructs. Seventeen percent of respondents also attributed “Research Activities” to supporting their identity and intent. For example, one student shared, “The process of learning python and dedicating my project to it was extremely empowering to me in feeling like I was accepted in the STEM world—especially knowing that the data analysis I was doing was similar to what actual scientists do.”

Survey data also showed that participants’ presentations and project work supported STEM learning across all constructs (Table 7). At the culmination of each experience, students created projects showcasing their learning. For more description on culminating projects and student quotes highlighting the positive impact of these projects, please see the Supplemental Material.

## Discussion

All young people are part of a STEM ecosystem that includes experiences in and out of school that influence their intent to pursue and persist in STEM degree programs. Participation in informal STEM learning has been shown to support sustained interest in STEM and STEM career paths, with potentially greater impact than formal experiences (P. Bell et al. 2009; Falk and Dierking, 2010; Gupta and Siegel, 2008; Habig et al. 2020; Tytler and Osborne, 2012; VanMeter-Adams et al. 2014). However, in science learning settings, learners from HMC are more likely to find a shift to full engagement in science learning difficult or inaccessible as they struggle to negotiate the valued practices and identities at play (Morton and Parsons, 2018). As a result, young people may not see science as relevant to their daily lives, feel welcome in science, or see science as something for them (Bang et al. 2013; Kang et al. 2019; Shea and Sandoval, 2020). The current study sought to identify practical insights for supporting equitable STEM learning by studying the design and implementation of a variety of STEM learning experiences provided by SEE. To do this, retrospective survey data collected from 56 alumni who had participated between 2003 and 2019 was analyzed, as well as retrospective pre-post survey data collected from 183 students who had participated in one of four SEE experiences between 2019 and 2021. The data collection tools were framed around common constructs considered in designing and implementing equitable STEM learning settings, and the analysis addressed three study questions.

### Q1: What progress on STEM pathways do students from HMC make as a result of participation in informal STEM learning experiences?

To address the first study question, a traditional view of STEM pathways was used to demonstrate that SEE alumni made progress along STEM pathways, including completing or intending to complete an undergraduate, graduate, and/or professional degree in a STEM major, as well as being actively or previously employed in a STEM field. Studies show that focusing on graduation rates as the defining factor in STEM success does not take into account the institutional context and student characteristics (National Academies of Sciences, Engineering, and Medicine, 2016). Therefore, the current study also took a broad view of STEM pathways, supported by a 2016 consensus report from the National Academies of Sciences, Engineering, and Medicine, that focuses on dimensions of success with an expansive definition of “thriving” that includes informed decision-making, awareness of career options, connections to the community, and examination of barriers to STEM opportunities (Engineering, N. A. of, & Engineering, and M. N. A. of S., 2016; Schreiner, 2010).

The data presented in Fig. 2 highlights the important work SEE, and other informal extracurricular activities are doing to support students from HMC in making progress toward STEM success outside the formal classroom. Specifically, students reported that their SEE experience uniquely supported them in making informed decisions about their course of study, understanding potential STEM career pathways, content and practices, and building awareness of societal connections with STEM. As documented in the literature, this focus on broadening access through consequential participation can provide opportunities for young people to engage in authentic STEM-based practices that crosscut between disciplines historically unavailable to them (National Research Council, 2012); explicitly contest historical narratives of who can do science and develop science-linked identities (H. Carlone, 2017; Rahm and Moore, 2016; Thompson, 2014); and make direct connections to their everyday lives and better understand ways they can use science to advocate for their communities (C. Garibay and Teasdale, 2019; Stromholt and Bell, 2018). The practical implications of these results are for informal STEM settings to continue to emphasize: (1) full engagement of students from HMC as they contribute to authentic STEM projects that are relevant to their lives and communities, (2) opportunities for students to share their contributions publicly such as on a website so they can be recognized by others for their contributions, (3) opportunities for mentors and students to stay connected to support students as they navigate barriers and decisions on courses of study, potential STEM career pathways, and societal connections of their current and future work. Search Institute’s Developmental Relationship Framework can guide ways of supporting students (Search Institute, 2020).

### Q2: What successes and challenges emerge when young people from HMC engage in authentic STEM experiences?

The second study question was addressed through analysis of both alumni data and participant data from students who had engaged in a SEE experience between 2019 and 2021. By measuring engagement, identity, social capital, 21st Century Learning Skills, awareness, and intent, the study identified the following successes and challenges when engaging young people from HMC in authentic STEM experiences.

#### Students’ STEM learning can successfully be supported with a variety of experience durations, with some limitations.

**Fig. 5:**
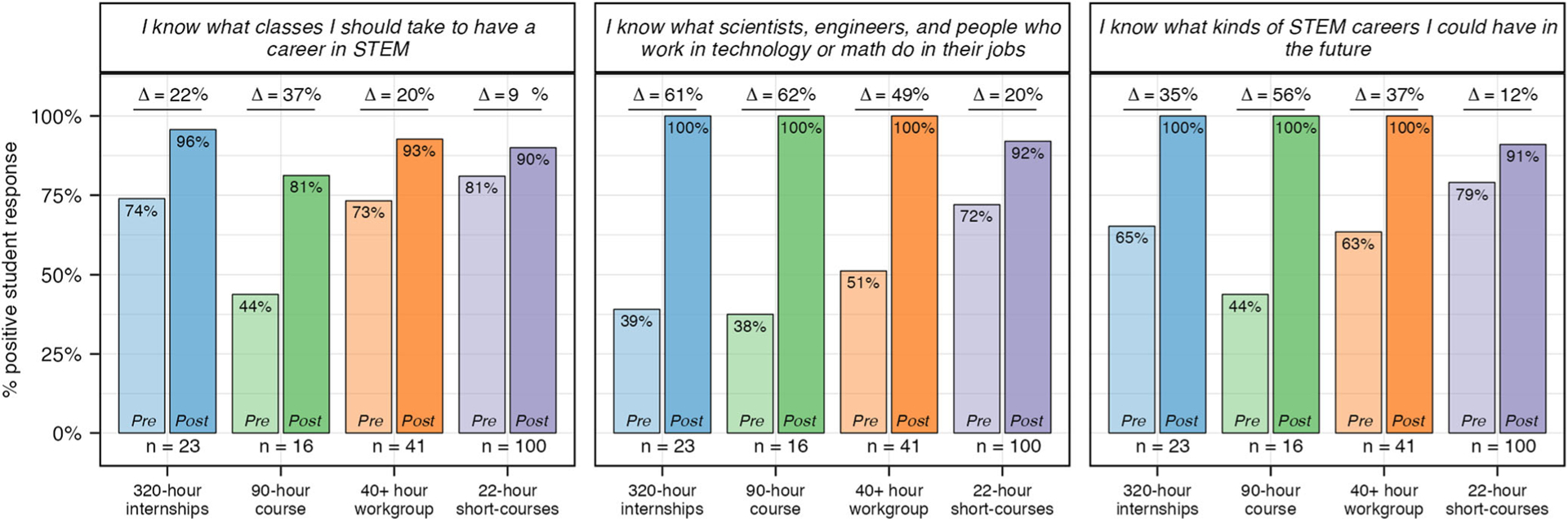
Pre and post survey results for questions related to awareness of STEM careers and pathways. Percent positive for each question related to STEM awareness was calculated for pre and post experience student responses. Students responded to each question using a 4-point Likert scale. “Strongly Agree” and “Agree” were coded as positive responses and used to calculate percent positive.

**Fig. 6:**
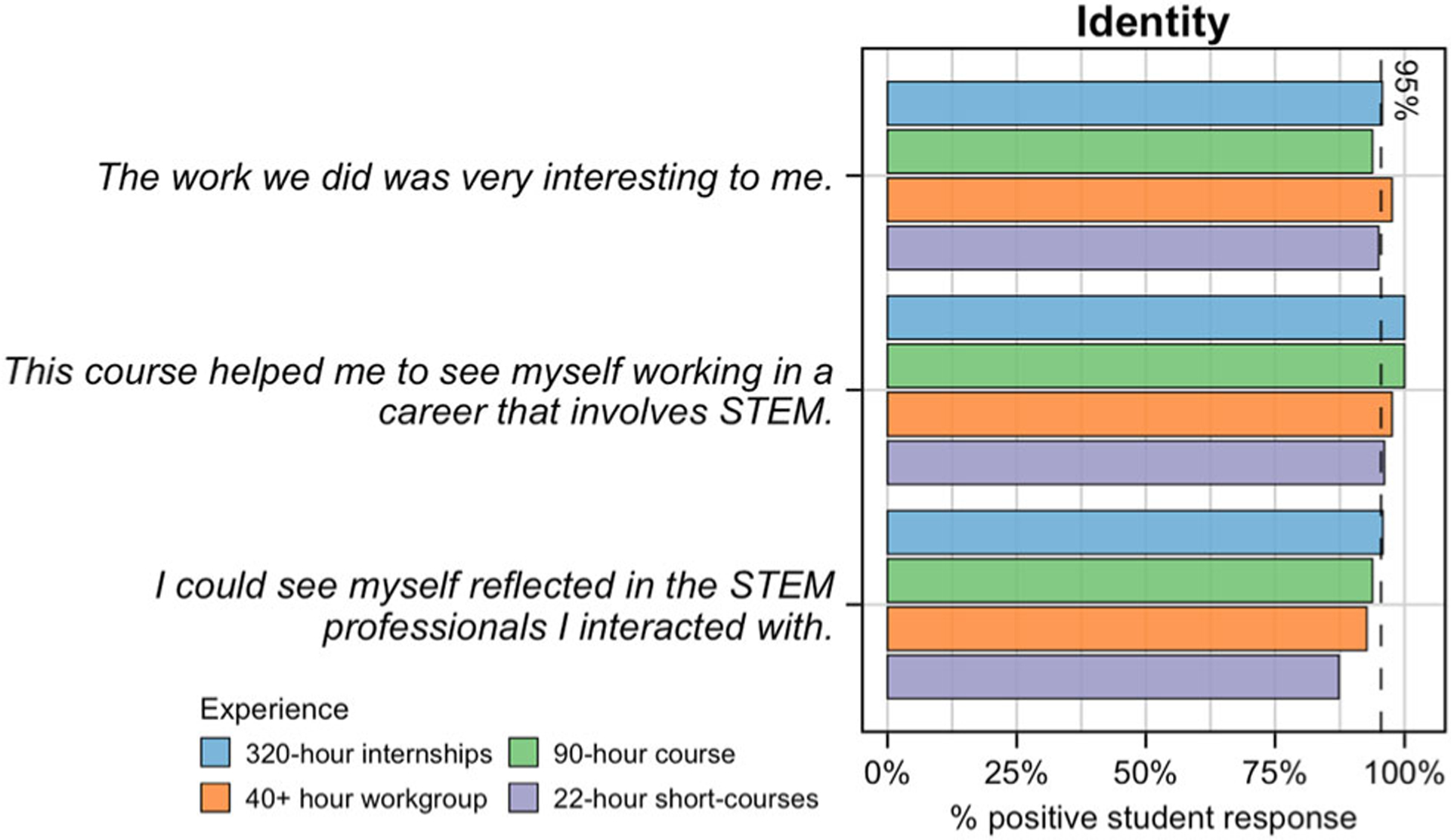
Post-only student survey results for questions related to STEM identity. Students responded to three post-only survey questions that serve to assess whether the experience provided a suitable environment for students to develop STEM Identity. Presented are the percentage of positive responses (“Strongly Agree” or “Agree”) for each question, color-coded by experience. The dashed line represents the average, which is 95% across all questions.

**Fig. 7:**
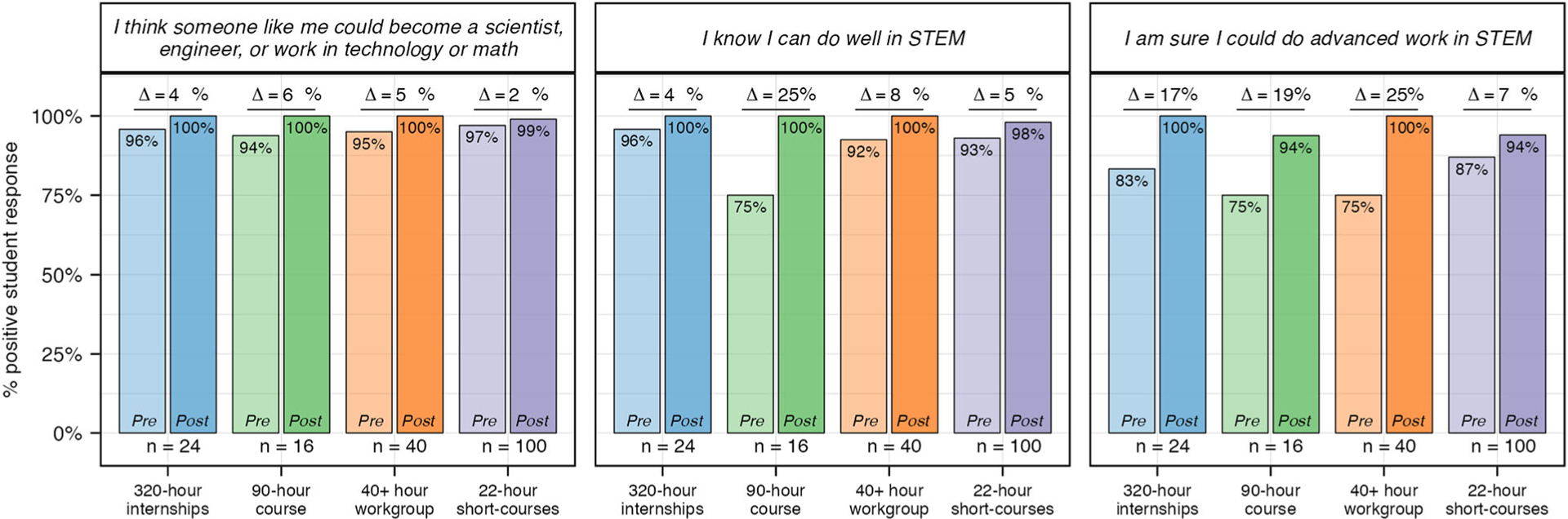
Pre and post experience student survey results for questions related to STEM identity. Percent positive for each question related to STEM identity was calculated for pre and post experience student responses. Students responded to each question using a 4-point Likert scale. “Strongly Agree” and “Agree” were coded as positive responses and used to calculate percent positive. *N* values: 320-h = 24; 90-h = 16; 40+ hour = 40; 22-h = 100.

**Fig. 8:**
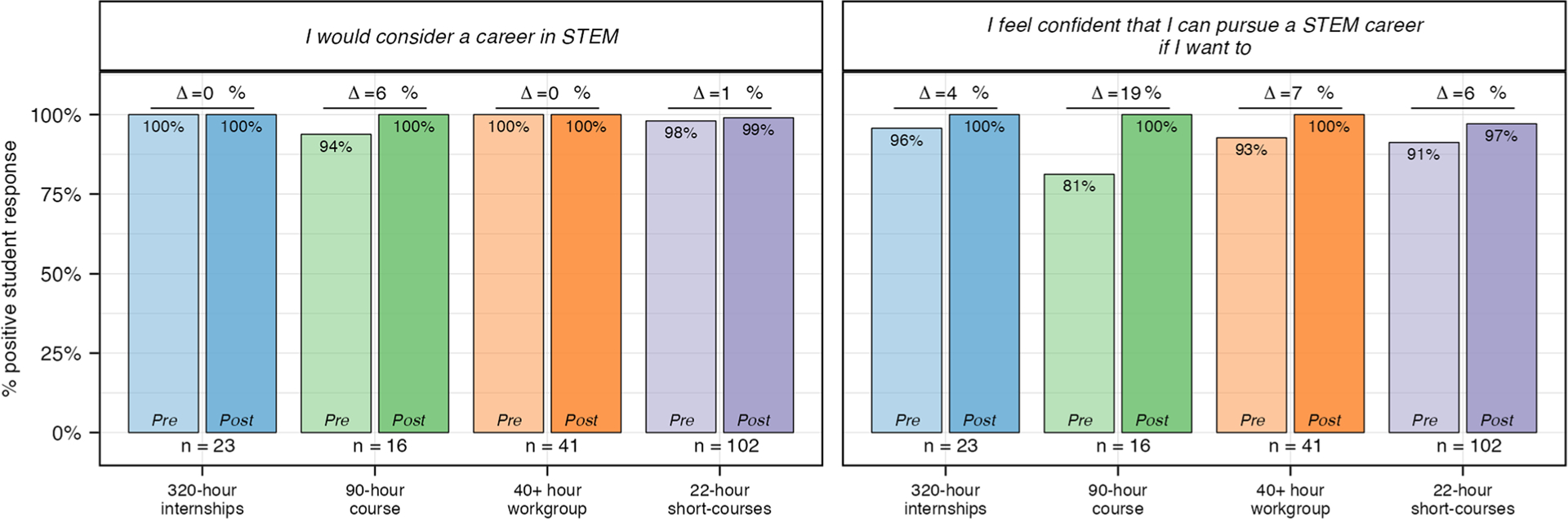
Pre and post survey results for questions related to intent to pursue a STEM career. Percent positive for each question related to intent to pursue a STEM career was calculated for pre and post experience student responses. Students responded to each question using a 4-point Likert scale. “Strongly Agree” and “Agree” were coded as positive responses and used to calculate percent positive. *N* values: 320-h = 23; 90-h = 16; 40+ hour = 41; 22-h = 102.

**Fig. 9:**
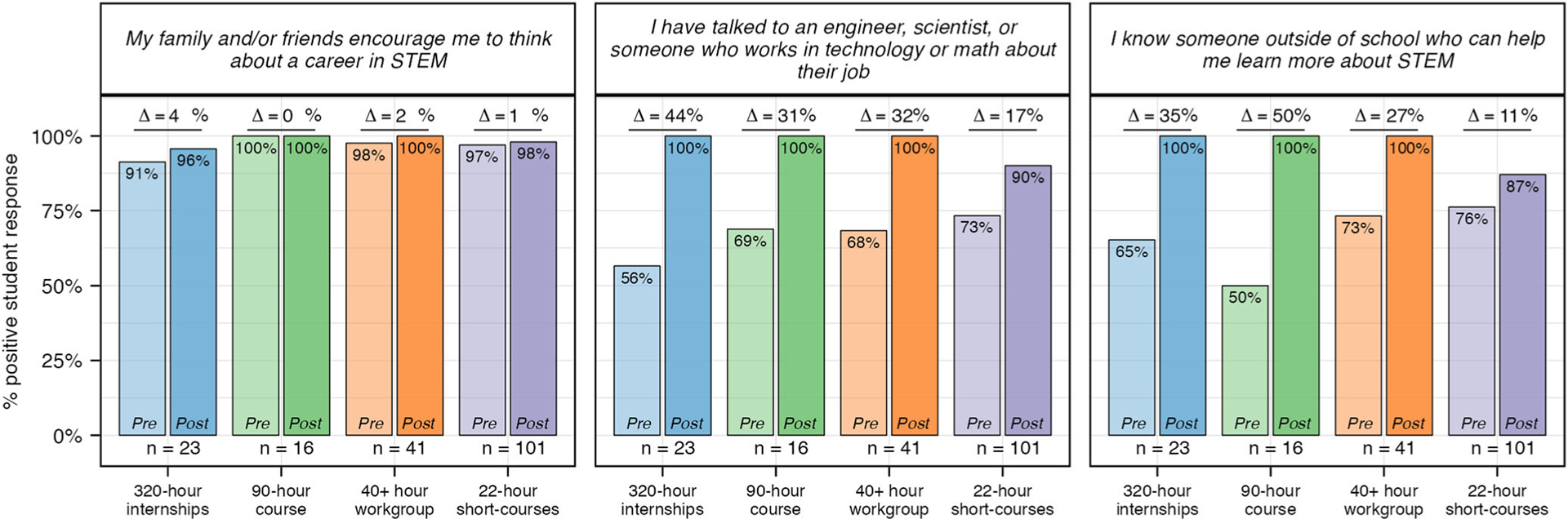
Pre and post experience student survey results for questions related to social capital. Percent positive was calculated for pre and post experience reflections across all students. Students responded to each question using a 4-point Likert scale. “Strongly Agree” and “Agree” were coded as positive responses and used to calculate percent positive. *N* values: 320-h = 23; 90-h = 16; 40+ hour = 41; 22-h = 101.

**Fig. 10:**
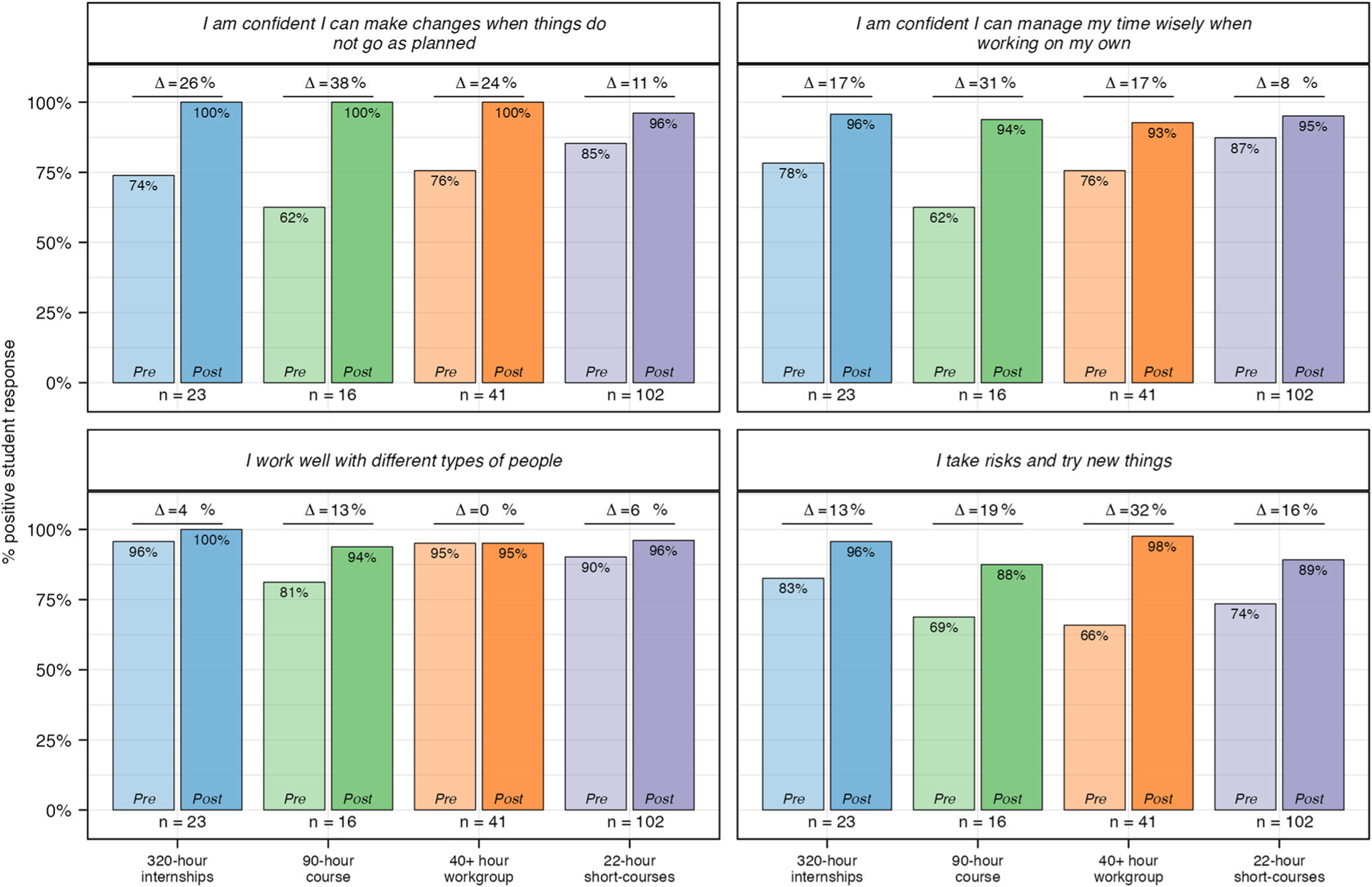
Pre and post student survey responses to questions related to 21st century skills. Percent positive was calculated for pre and post experience reflections across all students. Students responded to each question using a 4-point Likert scale. “Strongly Agree” and “Agree” were coded as positive responses and used to calculate percent positive. *N* values: 320-h = 23; 90-h = 16; 40+ hour = 41; 22-h = 102.

**Fig. 11:**
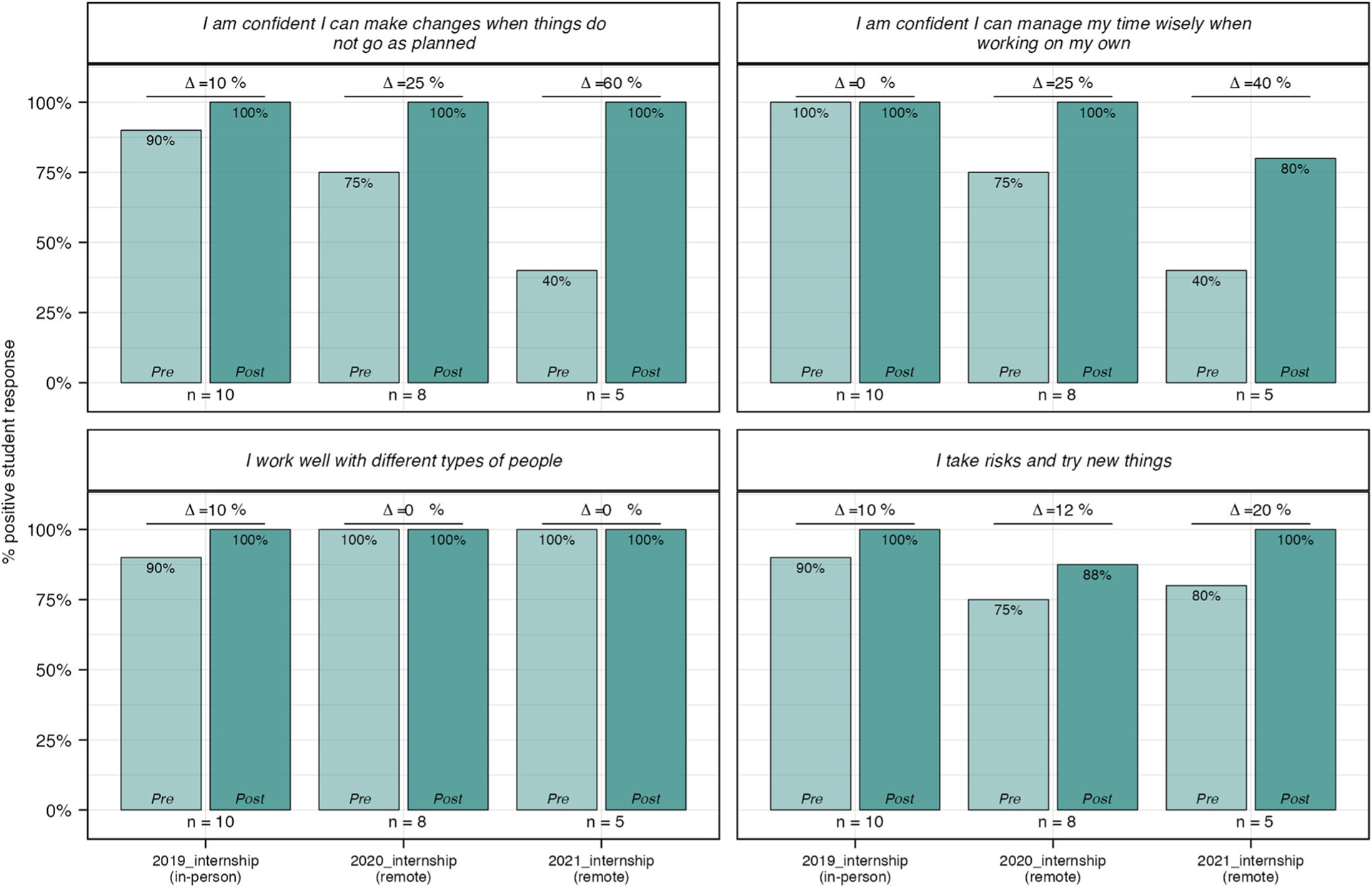
Similar outcomes in 21st century learning skills across remote and in-person SEE internship cohorts. Percent positive was calculated for pre and post internship reflections across all students. Students responded to each question using a 4-point Likert scale. “Strongly Agree” and “Agree” were coded as positive responses and used to calculate percent positive. IP = in-person experience; R = remote experience. *N* values: 2019 = 10; 2020 = 8; 2021 = 5.

The expansion of SEE to include 320-h, 90-h, 40+ hour, and 22-h programming provided a unique opportunity to explore how program duration may impact students’ STEM learning, particularly for students from HMC. As demonstrated from the data analysis presented in Figs. 2–11, the overall outcomes for all experiences were very similar, demonstrating the positive impact of SEE on supporting students’ success in STEM pathways regardless of the duration of the experience. Students who participated in any of the SEE experiences were consistently engaged during participation, developed awareness of STEM careers and pathways, built STEM identity, interest, social capital, and confidence to pursue a STEM career, and developed 21^st^ Century Learning Skills, regardless of the experience duration. This is positive news for programs with limited resources and / or programs interested in expanding their offerings. One of the many practical implications of this is to focus on co-creating programs using research-based design principles and common goals. The duration of the experience is less relevant if the design is focused on the principles and the co-created goals. Based on the large number of applications we receive from students from HMC, we are certain they are eager to participate. Positive outcomes are not only possible, but meaningful and long-lasting for participants. To increase the number of students from HMC applying to programs, focus recruitment efforts on partnering with other local high school programs that serve HMC, such as MESA, Upward Bound, and other such well-established programs. Additionally, provide stipends to enable participation. Finally, it is imperative to always follow through with programming at the highest level possible. Participants share their experiences with upcoming potential applicants. Positive experiences each year will lead to more and more applicants in future years.

One limitation, while subtle, emerged with the 22-h experience. Specifically, a few students reported that their 22-h experience did not support them in developing an understanding of “what kinds of STEM careers [they] could have in the future” and “what scientists, engineers, and people who work in technology or math do in their jobs” (*n* = 9 and 8, respectfully). This is in contrast to data from the 320-h, 90-h, and 40+ hour experiences in which 100% of students positively reported being supported in these areas (Fig. 5). Similarly, the results in Fig. 9 demonstrate fewer students from the 22-h short courses reported that they “have talked to an engineer, scientist, or someone who works in technology or math about their job” or “know someone outside of school who can help me learn about STEM.” Collectively, this data highlights the challenges of creating space in short-duration experiences for meaningful career-connected activities that align with students’ content learning. This, however, can be mitigated in many ways, such as by having scientists actively participate in programming through interviews, videos, meet-and-greets, job shadows, etc.

A second limitation is in the area of social capital. While social capital improved across all experiences, it is apparent in Table 5 that students’ connection to peers improves at a decreasing amount relative to the length of experience. We believe this is due, in part, to the amount of time and to cohort size. Students built capital with their mentors, but we did not include as much time for peers to connect, and that is apparent in the data. We also think this is due, in part, to the wording of the survey item. In a more recent survey, we asked students if they “plan to stay connected with one or more of the peers in my cohort” (rather than “to peers in my cohort.” This resulted in higher percentages of students answering “Agree” and “Strongly Agree.” This highlights the challenges of choosing what to focus on with the shorter amounts of program time. However, this can be easily mitigated if social capital with peers is an important learning goal. One of many practical ways to do this is to have a near-peer mentor join the cohort with the explicit goal of helping the cohort connect through team-building activities and common STEM-related projects. Use near-peer mentors who were previous participants if possible. This provides leadership skills for a near-peer mentor who may be just 1–2 years older than the cohort and provides concrete ways of having students spend time with each other while learning new content and skills.

#### Both in-person and remote informal STEM learning experiences can support students’ STEM success, with some limitations.

The data provided an opportunity to study whether the mode of experience (in-person vs remote) impacted students’ STEM learning, as the 320-h internship experience was held in-person in 2019 and then transitioned to remote for 2020 & 2021 due to COVID-19 restrictions. Based on the data analysis in Table 5, the mode of internship experience did not appear to have a significant impact on student growth. We are cautious to extend this claim to all experiences without having definitive case-control comparisons, as each type of experience is unique in content and delivery. As an example, one of the 22-h remote short courses required students to conduct a hands-on laboratory experiment at home. While supplies were provided, some students reported not being able to complete the experiment successfully due to issues in receiving materials and finding space in their homes to maintain an experiment. Despite these isolated challenges, there is still value in providing remote STEM experiences, as they provide opportunities to extend the reach of the program and access to students who may have time and location limitations. However, care should be taken to understand where each student is with their level of interest in the content area, as students who enter into experiences with established “individual interest” may be more resilient when faced with challenges than others who are in an earlier phase of interest development (Hidi and Renninger, 2006). A practical implication of this is for program providers to get to know students prior to and/or early in their participation. True co-creation of programming with students makes it possible to provide the care that is suggested above. It also allows you to craft an experience that has the appropriate level of risk and reward. Other studies of informal STEM learning experiences during COVID-related closures in the United States found that these types of programs could support STEM-related outcomes such as researcher identity (Marvasi et al. 2019; Ray and Srivastava, 2020). Another practical implication for improving researcher identity is to consider virtual labs that may be more effective than a challenging hands-on at-home lab without in-person support. Co-creation of the experience with students also fosters ongoing conversations to process the labs, content, and general ups and downs of research in a way that is supportive of researcher’s identity.

#### Supports are needed to help students understand and navigate barriers along STEM pathways and understand societal connections to STEM.

The student alumni data brought to light a need for both formal and informal programs to help students understand how to navigate the barriers they might face along their STEM pathway journey. Student alumni rated SEE, extra-curricular, and classroom experiences lower in this component as compared to the other components of STEM pathway success (Fig. 2). Additionally, alumni reported that SEE better supported their understanding of societal connections to STEM as compared to extracurricular and classroom experiences, they gave this component a lower average rating across all experiences. These are important insights that need to be addressed to ensure STEM learning opportunities are supporting students’ current and future success along STEM pathways.

Previous work has found that providing opportunities to approach STEM from a societal perspective (as well as from personal interests) is important to broadening their perspectives about STEM careers (Lee et al. 2018). Garibay (2015) found that these societal connections are especially important for students from HMC as they are more likely to view their purpose for majoring in STEM as a means to create a better world, concluding that “changing the culture of STEM disciplines to include and make these values more visible may go a long way in meeting the needs of [students from HMC] in STEM” (p. 627) (J. C. Garibay, 2015).

Areas of consideration for supporting students in understanding and navigating barriers and societal connections in STEM were identified through students’ open-response data (Table 4). Specifically, SEE alumni attributed their growth to “see[ing] many researchers in different roles and at different points in their careers” as well as “see[ing] how the science that I do could positively impact the world to a greater extent.” These student perspectives are supported by previous research demonstrating the importance of showcasing to students “a diverse range of identities” and “non-traditional ways of doing or being in STEM (Çolakoğlu et al. 2023). Recent studies have argued that there is a need to better understand and make explicit the barriers that students from HMC face as they navigate STEM pathways, such as exposure to stereotypes, inadequate academic preparation, and human and cultural capital (Hurtado et al. 2011; Major and Godwin, 2018; Pierszalowski et al. 2021). While making these barriers explicit to researchers and practitioners is crucial so that they can work to remove these barriers at the institutional level and reshape the culture of science, it is important for students to also be aware of barriers so that they can make choices that may enhance their experiences and socialization into STEM communities (e.g., Hurtado et al. 2011).

#### Students need support in making intentional connections to program goals.

Creating an environment in which students feel supported, comfortable in asking questions and sharing ideas, and understanding the goals and expectations, is critically important to their success during an experience as well as to the continued development of their interests in STEM (Hidi and Renninger, 2006). Because of this, students’ engagement can influence how they think about the impact of an experience on their STEM awareness, identity, interest, social capital, and 21st Century Learning Skills. In the present study, students reported being strongly engaged during their experience. However analysis of the data presented in Fig. 3 demonstrates students in the 90-h course rated their understanding of the goals lower than all other experiences. While this did not appear to impact the ratings of all other constructs for this group of students (Fig. 4), it is an important facet to call out as research strongly demonstrates the importance of goal-setting for “self-regulated learning, self-efficacy, intrinsic motivation, and cognitive engagement (Midwest Comprehensive Center, 2018). Additionally, it should be noted that efforts to support students in making connections to goals throughout the learning experience (not just at the beginning) are needed to support all phases of interest development (Hidi and Renninger, 2006). A practical implication that helps students make this connection is to create shared spreadsheets or program management charts. From the beginning and throughout their experience, participants can use these to track their progress toward achieving goals, subgoals, and milestones. This also builds students’ general, applicable professional job skills. A similar technique that further enhances STEM skills in the area of data visualization is to have them track goal progression by creating a heatmap (C. M. Ludwig, 2023).

### Q3: What aspects of informal STEM learning experiences support young people from historically marginalized communities in making progress on STEM pathways?

The third study question was addressed by analyzing open-response data from all SEE participants who were asked to reflect on their experience and identify specific aspects that contributed to their STEM pathway success.

#### Interactions with STEM professionals support students in making important connections to successful STEM career trajectories.

A common thread throughout the student responses was the positive impact of direct and indirect interactions with scientists and other STEM professionals from diverse STEM disciplines. SEE provided multiple and varied opportunities for students to learn about the experiences of professionals from diverse STEM fields and career paths, including question and answer sessions, exploring the suite of career-connected “Systems Thinkers in STEM” videos and profiles, and interviewing STEM professionals (Systems Education Experiences, 2023c). In addition, student interns also had the opportunity to attend authentic research presentations through their lab groups and at ISB-wide events. Alumni shared that these interactions introduced them to STEM fields and career pathways they were not aware of prior to their experience, and positive, focused, and respectful guidance by mentors supported them in building confidence (Table 7). Student participants echoed these statements by connecting their interactions with STEM professionals to the development of awareness, intent, social capital, identity and 21^st^ Century Learning Skills (Table 7). One student shared that interviewing professionals helped them “become more aware of career options, but hearing about their journey and what exactly they did gave me a clear picture of what it takes to be successful.” Another student shared that one of the most important things they learning about STEM careers during their experience with SEE was how “numerous and accessible” STEM careers were and that there are “many different pathways to a career in the STEM field.” Students also reported that professionals helped them in identifying the next steps by “suggesting that we take the STEM classes that we can, and take time on our own to do what isn’t taught in class.”

These professional connections also expanded students’ social capital, extending the reach of the program beyond the boundaries of the experience. One student stated that “learning about each individual role was so cool and I know there is someone I can email if I have a question in a certain topic just because of how diverse the scientific focus is at ISB.” These examples and impact may come as no surprise based on the vast research on legitimate peripheral participation in communities of practice (Bloch et al. 1994; Clarke et al. 2001).

Though Gamse et al. (2017) found few studies that describe specifically how these mentor interactions lead to positive outcomes in undergraduate research experiences (Gamse et al. 2017), many informal STEM studies have shown that interactions with faculty/staff/STEM professionals are an important aspect of a meaningful learning community for students: for helping students to choose research projects (Craney et al. 2011), providing insights into STEM pathways and building social capital (Carrino and Gerace, 2016), and supporting students’ sense of belonging (Vannier et al. 2023). Opportunities to connect with STEM professionals are more readily available in informal STEM learning settings as they are typically situated within a professional STEM environment in which students have access to resources, space, people, and narratives that are generally unavailable to young people in formal classrooms. Results from the current study demonstrate that despite this access, intentional supports are needed to connected students to these resources. Analysis in 2023 completed by Çolakoğlu and colleagues suggests that in addition to professional engagements with STEM professionals from diverse backgrounds, programs should intentionally build in regular social activities “that allow students to get to know each other as well as educators and community members on a personal level” (Çolakoğlu et al. 2023). These activities serve to “foster social networks between participants as well as between participants and staff.” Having a supportive community of friends and mentors provides diverse perspective and guidance to help individuals navigate pathways and expand opportunities.

#### Authentic research activities provide opportunities for using professional tools and foster positive identities.

Interest and engagement with STEM and STEM-related careers are linked to how young people consider themselves in relation to narratives of “what it means to be a science person” (Kang et al. 2019). Therefore, STEM identity is an integral aspect of learning that requires individuals to engage in appropriate practices that help them negotiate the tension between their everyday ways of being with ways of being in science communities (H. B. Carlone and Johnson, 2007; Holland et al. 1999). As part of each SEE experience, students built their understanding of phenomena and identified explanations and/or solutions to driving questions. In doing so they were guided through authentic STEM research practices, used authentic STEM tools and resources, and built knowledge of core STEM principles. Student reflections highlight the impact of these authentic experiences in developing their identity as a person in STEM and in building confidence in their knowledge and skills. These findings align with other studies of student engagement in authentic STEM activities (Estrada et al. 2018; Singer et al. 2020; Talafian et al. 2019). For example, Newell and Ulrich found that authentic research activities in course-based undergraduate research experiences led to positive outcomes such as scientific self-efficacy, scientific identity, career intent, value orientation, and mentorship (Newell and Ulrich, 2022).

## Conclusion

There continues to be an important need within the STEM education community for researchers and informal science educators to provide equitable STEM experiences for students from HMC to participate in addressing the complex global issues our communities face. This study identifies consequential insights for supporting students from HMC in making progress on STEM pathways and demonstrates that informal STEM learning programs can provide experiences that are unique from formal education settings. These real-world experiences and environments uniquely supported students in making informed decisions about their course of study, understanding potential STEM career pathways, content and practices, and building awareness of societal connections with STEM. Participant data demonstrate that though students may need more explicit support to make connections to program goals, interactions with STEM professionals supported students in making important connections to successful STEM career trajectories, and authentic research activities provided opportunities for using professional tools and fostering positive identities. Importantly, this study demonstrates that these impacts can be achieved in longer-duration internships, intensive 90-h courses, medium-duration workgroups, and shorter-duration courses, both in-person and in virtual settings, with some trade-offs. Finally, this study shows that further supports are needed in these settings to help students understand and navigate barriers along STEM pathways and understand societal connections to STEM.

This study provides practical insights for advancing informal STEM learning and outcomes for students from HMC. When considering what efforts or programs to lead and sustain, there are ample program models that align with available resources and capacity. New modes and inclusive cultures of virtual programming have opened many doors for students and practitioners. This study demonstrates that leading and sustaining these effective STEM programs is possible with minimal resources and leads to outcomes that have been shown to be important in students’ ongoing STEM journeys. The analyses also identified meaningful program components and ways to mitigate trade-offs when scaling STEM experiences to help ensure positive STEM trajectories. These include providing program management charts, near-peer mentors, and staying true to the co-creation of all experiences to facilitate honest, two-way, supportive discussions. Ultimately, this study provides evidence that wide integration of such programs has the potential to address societal challenges by (1) broadening the STEM workforce towards having a more representative number of people from historically marginalized communities in STEM fields, (2) improving the likelihood of success in STEM for these communities, (3) enhancing real-world problem solving and innovation, and (4) improving society’s overall readiness to benefit from today’s and tomorrow’s STEM advances.

## Supplementary Material

Supplementary Tables & Figures

## Figures and Tables

**Table 1:** The six constructs that were used to measure and analyze outcomes for students from HMCs who participated in hands-on STEM experiences.

Construct	Short description
Engagement	How students are involved in their learning
Awareness and Intent	Awareness of and movement towards STEM degrees and professional levels. Both of these overlapping constructs are part of a person’s STEM Pathway.
Identity	How students perceive themselves in relation to what it means to be a “science person” Dynamic, social, and situational Emerges from an ongoing process of performance, recognition & positioning by others
Social capital	A broad, overlapping construct that includes the set of intangible resources, from their interpersonal relationships or social institutions that lead to productive advances that would otherwise be unlikely.
21st Century Learning Skills	The set of professional skills, such as critical thinking and collaboration, that are considered necessary for success in project future workforces

These constructs were chosen based on their ability to provide consequential insight into i) whether or not participants were able to advance their STEM learning and outcomes, and ii) what aspects of their experience supported them in developing STEM literacy and sustaining interest and participation in STEM.

**Table 2:** a: Overview of the four STEM experience models. b: Relative emphasis placed on each program design principle within each STEM experience model.

a				
STEM Experiences:	320-hour internship	90-hour course	40+ hour workgroup	22-hour short courses
**Formats:**	In-person (2019) Remote (2020 & 2021)	In-person (2019)	Remote (2020)	Remote (2021)
**Participants:**	24 rising seniors	17 rising juniors & seniors	42 rising seniors only	180 rising juniors, rising seniors, & recently graduated seniors
**Total Survey Responses**	n = 24	n = 16	n = 41	n = 102
**Purpose:**	Cultivate interdisciplinary skills for solving complex problems while broadening and diversifying the STEM workforce			
**Goals:**	**Learn** about systems, **Develop** professional skills, **Explore** new topics, **Collaborate** on team projects, **Network** socially and professionally			
**Activities Summary:**	Directly involved in systems biology research projects Translated systems research into open-access curriculum modules (optional) Developed website to showcase learning	Completed lessons and human cadaveric specimen dissections exploring systems approaches in health and medicine Learning showcased with art projects presented at an in-person community event and featured on the course website	Designed and completed computational modeling projects to understand and solve systems problems Projects showcased at an online community event and featured on the course website	Completed 2 hours of learning activities about systems, systems modeling, and developing systems thinking skills (Tier 1) Completed 20-hours of activities through existing program curriculum modules (Tier 2)
**Examples of How the Design Principles Specifically Support Students from HMC**	Participants had never before contributed to a professional STEM project. By the end of the experience, each student had navigated the ups and downs of real-world systems research & created & launched a webpage detailing their project, their profile, and what they learned. This was used to secure entry into college and to succeed in future STEM experiences.	Participants worked within multiple professional settings & learned about careers & innovative training modes that help people gain new skills quickly. They also used art to share findings in a community event & online. New confidence, awareness & skills were used to envision & reach for new experiences.	Participants took risks to learn computational skills during the pandemic – something they thought they were not interested in and could not do. They learned from professionals from HMC & shifted to full engagement. This all led to new career options, confidence, an online presence that showcased their skills and who they are, and opened new doors.	Participants were brought together online from across the world to learn from scientists from HMC and to connect with other youth who shared STEM interests. This enhanced social capital, career awareness & provided innovative research skills that were featured online. This helped youth see science as relevant to their daily lives.
b				
STEM Experiences (to the right) Program Design Principle (below):	320-hour internship	90-hour course	40+ hour workgroup	22-hour short courses
Provide opportunities for young people to make meaningful contributions to professional projects.				
Set young people up to take risks, get supportive feedback, and solve problems.				
Support youth explicitly to navigate unreasonable or institutionalized barriers they or others may face based on race/ethnicity/gender.				
Provide multiple and varied opportunities for professionals to share their personal stories and STEM pathways with young people, especially those who might share aspects of their cultural experiences, discourses, and values.				
Make explicit the circuitous nature of STEM pathways.				
Provide access to technology and other resources otherwise unavailable.				
Cultivate a supportive community (both cohort and mentors).				
Support young people to create a professional presence and learn about networking.				
Engage families in supporting young people in STEM pathways.				
Emphasize the innovative and interdisciplinary approaches and positions that young people may not learn about elsewhere.				

a: All experiences were co-created by scientists, educators, and students using the same set of research-based agreed upon design principles. All experiences also shared the same overall goals. The context, timeline, and content varied based on students’ interest and the connected research projects. Rising seniors participated during the summer between their 11th and 12th grade years and were most often 17 years old; rising juniors between their 10th and 11th grade years and were most often 16 years old. The Total Survey Responses (n values) represent the number of people who completed either some or all of the survey items. Depending on the cohort, 71–94% of participants were students from HMC.

b: This heat map displays the relative emphasis placed on each of the 10 shared design principles across all four STEM experience models. Each STEM experience model was co-created with students, educators, scientists, and other pertinent stakeholders (e.g., physicians, artists, software engineers, etc.) based on 10 program design principles. Due to time availability, content, and specific experience objectives, the level each design principle was emphasized varied within each model. This heat map demonstrates the relative level emphasized for each program design principle on a scale from low with the lightest gray to high with the darkest gray.

**Table 3:** Overview of the participant survey format.

Construct	Item format	Number of items
Engagement (satisfaction & interest)	Post only	7
Awareness	Pre/Post	3
Identity	Post only, Pre/Post	3, 3
Intent	Pre/Post	2
Social capital	Post only, Pre/Post	2, 3
21st century learning skills	Pre/Post	4

Student survey items aligned with one of six constructs. Engagement was subdivided into items related to “satisfaction” (4 items) and “interest” (3 items), all of which were post-only format. Identity and Social Capital items were in both post-only and pre-post formats. Awareness, Intent, and 21st Century Learning Skills were provided in the pre-post format only.

**Table 4:** A representative selection of student intern alumni responses.

Pathway component	Intern alumni student responses
Course of study	“My time at ISB opened my eyes to the diversity of degrees in biological research. I came in with the misconception that people who do biology research have degrees in biology. I was surprised to find there were scientists with degrees in math, chemical engineering, and physics, and this has shaped the way I think about majors.” (2017 Intern) “The internship was very influential in deciding my college major: I barely knew what bioinformatics was before it, but now I plan to apply for it.” (2019 Intern)
Career pathways	“The mentors I got to interview came from a multitude of backgrounds and experiences that opened my eyes to what potential pathways I could pursue. I also was able to see how broad STEM careers could be and how many different paths I could take to pursue any given career.” (2018 Intern) “My internship at ISB was one of the reasons why I chose to pursue Microbiology. Working there I realized that there were so many different career options outside of just medicine. I learnt a lot from interviewing the researchers and attending talks.” (2016 Intern)
Content & practices	“This program truly gives students a ‘career-launching starter kit.’ I learned how to pipette, use a centrifuge, manipulate data in Excel, keep a lab notebook, and so much more that I use in college constantly. Moreover, I learned how to read a scientific paper which has been an invaluable skill in university courses.” (2017 Intern). “It was one of the best experiences I had to prepare me for proper lab etiquette. It also was a safe place to step outside my comfort zone.” (2015 Intern) “I also really appreciated being able to sit in on presentations by faculty/staff at ISB; I observed a lot of analytical/critical audiences responding to preliminary data which is an important exchange to understand within research.” (2013 Intern) “I think one of the biggest impacts was having the experience of WORKING. Understanding what being in a lab might be like, what waking up every day and going into work is like.” (2013 Intern)
Barriers	“It was helpful to see many researchers in different roles and at different points in their careers and illuminated the different barriers and career options in the STEM field.” (2018 Intern)
Societal connections	“Definitely got to see how the research being done at ISB could go on to practically help people, clearer societal motivation than with most other STEM experiences I had.” (2010 Intern) “I got to see how the science that I do could positively impact the world to a great extent and the plethora of options that are available for me to pursue.” (2019 Intern)
General	“This experience provided one-on-one mentorship which helped the guidance be more specific to my interests.” (2019 Intern) “My ISB experience helped me feel confident in pursuing an undergraduate degree in biological sciences since I had a positive internship experience.” (2013 Intern) “I was treated with a great deal of respect at ISB. My mentors were patient and generous with their time. It made me feel like being a scientist was really possible. I think the experience grew my confidence.” (2012 Intern)

This and other responses demonstrate progression through STEM pathway components. Overall, their experience was positively impacted by ISB’s research environment, and authentic hands-on experiences. More quotes can be found in the Supplemental Material. Based on survey responses, 80% or more of the students who responded to the survey were people from HMC.

**Table 5:** Post-only student survey results to highlight the percentage of SEE students planning to stay connected to mentors and peers.

I plan to stay connected…	320-h internships	90-h course	40+ hour workgroup	22-h short-courses
…with one or more of my mentors.	100%	100%	88%	83%
…to peers in my cohort.	100%	94%	85%	56%

Percent positive was calculated for post-only experience reflections across all students. Students responded to each question using a 4-point Likert scale. “Strongly Agree” and “Agree” were coded as positive responses and used to calculate percent positive.

**Table 6:** SEE successfully supports STEM learning regardless of mode (in-person vs remote).

	2019 (IP)	2020 (R)	2021 (R)
Awareness	2.7, 3.7 (38%)	2.5, 3.7 (48%)	2.3, 3.4 (49%)
Identity	^#^3.5, 3.8 (8%)	3.3, 4.0 (20%)	^#^2.9, 3.8 (33%)
Intent	3.7, 4.0 (10%)	3.7, 4.0 (8%)	3.1, 4.0 (29%)
Social Capital	3.0, 3.8 (26%)	3.0, 3.8 (28%)	2.9, 3.9 (34%)
21st CLS	^#^3.3, *3.7 (11%)	2.9, ^3.8 (29%)	#2.7, *^3.3 (22%)

Mean values were calculated for pre and post student responses for each internship cohort; IP = in-person experience; R = remote experience. Values are formatted as pre, post (percent change). Two-sample unequal variance *t* test was performed on pre and post values to evaluate significance between each cohort. Significant difference was calculated for pre values between 2019 & 2021 cohorts (#*p* = 0.02 for Identity; 0.01 for 21st CLS); and for post values between 2019 & 2021 cohorts (*p = 0.02 for 21st CLS), and between 2020 & 2021 cohorts (^*p* = 0.03 for 21st CLS).

**Table 7:** Themes in students’ open responses to survey items regarding activities that support their development within each construct.

Activity themes	Supported constructs (% of students who attributed each theme to a supported construct)
Research activities	Identity (17%) Intent (17%)
Using scientific tools and materials	Identity (26%) Intent (17%)
Discussing primary literature	Identity (10%) Intent (7%)
Collaborating with peers and group work	21st Century Learning Skills (47%) Social Capital (22%) Intent (4%) Identity (3%)
Presentations and project work	Social Capital (24%) 21^st^ Century Learning Skills (14%) Identity (10%) Awareness (7%) Intent (7%)
Guest speakers, interviews, and career-connected videos	Awareness (72%) Intent (29%) Social Capital (20%) Identity (9%) 21st Century Learning Skills (5%)

Student participants were asked to share which activities supported their development in each of the five constructs. These open responses were coded based on emergent themes across all experiences 2019–2021. The percent of students who attributed each theme to a supported construct was calculated based on the total number of respondents for each respective construct (*n* = 95 for Awareness, 145 for Identity, 129 for Intent, 132 for Social Capital, and 116 for 21st Century Learning Skills (21st CLS)). Not all students provided responses for each construct.

## Data Availability

The datasets generated and/or analyzed during this study are not publicly available due to general data protection regulations, but are available from the corresponding authors on reasonable request. The curricular materials, program overviews and design principles are available publicly on the SEE websites (Systems Education Experiences, 2023b). More detailed program frameworks, agendas, and course materials are available from the corresponding authors upon reasonable request.
