# Supplementary material for: Consequential insights for advancing informal STEM learning and outcomes for students from historically marginalized communities
Source: Humanit Soc Sci Commun. Author manuscript; Available in PMC 2024 Jul 19. (PMC11259043; doi:10.1057/s41599-024-02797-w)
Supplement: Supplementary Tables & Figures [file NIHMS2008614-supplement-Supplementary_Tables___Figures.pdf]

SUPPLEMENTAL INFORMATION

Consequential insights for advancing informal STEM learning and outcomes for students from historically marginalized communities

Claudia McLaughlin Ludwig[1]*, Rebecca A. Howsmon[1,7], Shelley Stromholt[5], Jacob J. Valenzuela[1], Rachel Calder[1,6], and Nitin S. Baliga[1,2,3,4]*

[1]Institute for Systems Biology, Seattle, WA, USA
[2]Departments of Biology and Microbiology, University of Washington, Seattle, WA, USA
[3]Molecular and Cellular Biology Program, University of Washington, Seattle, WA, USA
[4]Lawrence Berkeley National Lab, Berkeley, CA, USA
[5]Aspect Research + Evaluation, Seattle, WA USA
[6]OMNY Health, Atlanta, GA USA
[7]Ballard High School, Seattle Public Schools, Seattle, WA USA

*Corresponding Authors

Correspondence to Claudia McLaughlin Ludwig: cludwig@isbscience.org

Correspondence to Nitin S. Baliga: nbaliga@isbscience.org

Institute for Systems Biology (ISB)

401 Terry Ave N, Seattle, WA 98109

T: 206.732.1453 (CML) and 206.732.1266 (NSB)

The following pages contain **Supplemental Text and Data** that provides more information for the study shared in *Consequential insights for advancing informal STEM learning and outcomes for students from historically marginalized communities*. In text citations are listed in the main paper's references section. Additional websites are listed to provide more information within this supplemental text.

Supplemental Table 1: Participant Data Demographics in Relation to Gender. The percent of student respondents who identify as female, male or non-binary is provided for each experience. Students self-identified gender on the retrospective impact survey taken at the completion of their experience. Completion of the survey was optional and students were not required to respond to every survey question, therefore the number of survey responses (n) for each program differs from the total number of participants. Of the 262 students who participated in one of the four experiences, 183 (70%) completed all or part of the survey; 153 responded to the question on gender identity with 74% identifying as female and 1% identifying as non-binary. This aligns with our application data in which 77% of the students accepted into these four experiences identified with one or more communities that have been historically marginalized in STEM. Students also shared with staff during programming and/or after programming how they identify which was recorded by staff and included in program statistics. We report these numbers in this table in the "*Total Number Historically Marginalized as shared by participants with staff" column.

**2019-2021 Program**	**Total participants**	***Total # Historically Marginalized in STEM as shared by participants with staff**	**Total survey responses**	**Self-identified on the Retrospective Survey**			**Prefer not to respond or no response** n (% of total participants)
				**Female** n (% of survey responses)	**Male** n (% of survey responses)	**Non-binary** n (%of survey responses)	
320-hour internship	23	20 (87%)	19	15 (79%)	4 (21%)	0 (0%)	4 (17% of tota)
90-hour course	17	16 (94%)	14	13 (93%)	1 (7%)	0 (0%)	3 (18%)
40+ hour workgroup	42	30 (71%)	36	24 (67%)	12 (33%)	0 (0%)	6 (14%)
22-hour short courses	180	136 (76%)	84	61 (73%)	22 (26%)	1 (1%)	96 (53%)
TOTAL	262	202 (77%)	153	113 (74%)	39 (25%)	1 (1%)	109 (42%)

Supplemental Table 2: Participant Data Demographics in Relation to Race/Ethnicity. The percent of student respondents who identify with the races/ethnicities in the top row is provided for each program. Students self-identified race/ethnicity on the retrospective impact survey taken at the completion of their experience. Completion of the survey was optional and students were not required to respond to every survey question, therefore the number of survey responses (n) for each program differs from the total number of participants. Due to rounding and small n-values for each category, the percentages do not add to 100%. Of the 262 students who participated in one of the four experiences, 183 (70%) completed all or part of the survey; 99 responded to the question on race/ethnicity. The self-report numbers align with our application data in which 77% of the students accepted into these four experiences identified with one or more communities that have been historically marginalized in STEM. Students also shared with staff during the programming and/or after programming how they identify which was recorded by staff and included in program statistics. We report these numbers in this table in the "*Total Number Historically Marginalized as shared by participants with staff" column. Please also note that 81.7% self-identified via the Retrospective Survey as BIPOC (Black, Indigenous, People of Color). However, not all participants who identify as BIPOC are historically marginalized or currently minoritized in STEM and are therefore not the focus of this study. ^Also note that we did have participants who identify as being part of Indigenous communities. They are not included in a distinct column in this table because these participants identified themselves as having more than one race / ethnicity in all cases and as such are included in that column with the ^.

**2019-2021 Program**	**Total participants**	***Total # Historically Marginalized in STEM as shared by participants with staff**	**Total survey responses**	**Self-identified on the Retrospective Survey**								**Prefer not to respond or no response** n (% of total participants)
				**African** n (% of survey responses)	**African American** n (% of survey responses)	**Asian** n (% of survey responses)	**Asian Indian / Indian** n (% of survey responses)	**Hispanic or Latinx/a/o** n (% of survey responses)	**Middle Eastern** n (% of survey responses)	**^Identify with more than one race / ethnicity** n (% of survey responses)	**White or Caucasian** n (% of survey responses)	
320-hour internship	23	20 (87%)	18	0 (0%)	2 (11.1%)	5 (27.8%)	3 (16.7%)	0 (0%)	1 (5.6%)	6 (33.3%)	1 (5.6%)	5 (21.7%)
90-hour course	17	16 (94%)	14	0 (0%)	0 (0%)	2 (14.3%)	7 (50.0%)	1 (7.1%)	0 (0%)	1 (7.1%)	3 (21.4%)	3 (17.6%)
40+ hour workgroup	42	30 (71%)	35	0 (0%)	1 (2.9%)	13 (37.1%)	11 (31.4%)	1 (2.9%)	0 (0%)	2 (5.7%)	7 (20.0%)	7 (16.7%)
22-hour short courses	180	136 (76%)	32	1 (3.1%)	0 (0%)	16 (50.0%)	0 (0%)	1 (3.1%)	1 (3.1%)	6 (18.7%)	7 (21.9%)	148 (82.2%)
TOTAL	262	202 (77%)	99	1 (1.0%)	3 (3.0%)	36 (36.4%)	21 (21.2%)	3 (3.0%)	2 (2.0%)	15 (15.1%)	18 (18.2%)	163 (62.2%)

Supplemental Table 3 with Heat Map Figure: Mean pre and post values for each experience. Mean values were calculated for pre and post student responses within each experience. Values are formatted as pre, post (point difference between pre and post). The heat map visualizes these numbers with blue being used as the lowest difference (0.2) and yellow as the highest difference (1.1). All differences are positive showing an increase across all constructs.

	320-hour internships	90-hour course	40+ hour workgroup	22-hour short-courses
**Awareness**	2.5, 3.6 (1.1)	2.4, 3.4 (1.0)	2.6, 3.4 (0.8)	3.1, 3.4 (0.3)
**Identity**	3.3, 3.9 (0.6)	3.1, 3.8 (0.7)	3.3, 3.7 (0.4)	3.4, 3.6 (0.2)
**Intent**	3.5, 4.0 (0.5)	3.4, 3.7 (0.3)	3.6, 3.8 (0.2)	3.5, 3.8 (0.3)
**Social Capital**	3.0, 3.9 (0.9)	2.9, 3.8 (0.9)	3.2, 3.7 (0.5)	3.2, 3.5 (0.3)
**21st CLS**	3.0, 3.6 (0.6)	2.9, 3.4 (0.5)	3.0, 3.4 (0.4)	3.0, 3.3 (0.3)

	320-hour internships	90-hour course	40+ hour workgroup	22-hour short-courses
**Awareness**	1.1	1.0	0.8	0.3
**Identity**	0.6	0.7	0.4	0.2
**Intent**	0.5	0.3	0.2	0.3
**Social Capital**	0.9	0.9	0.5	0.3
**21st CLS**	0.6	0.5	0.4	0.3

Supplemental Figure 1 – This line chart provides an alternative view, from the circular bar chart in Figure 4B, that summarizes the percent change for each construct for each program.

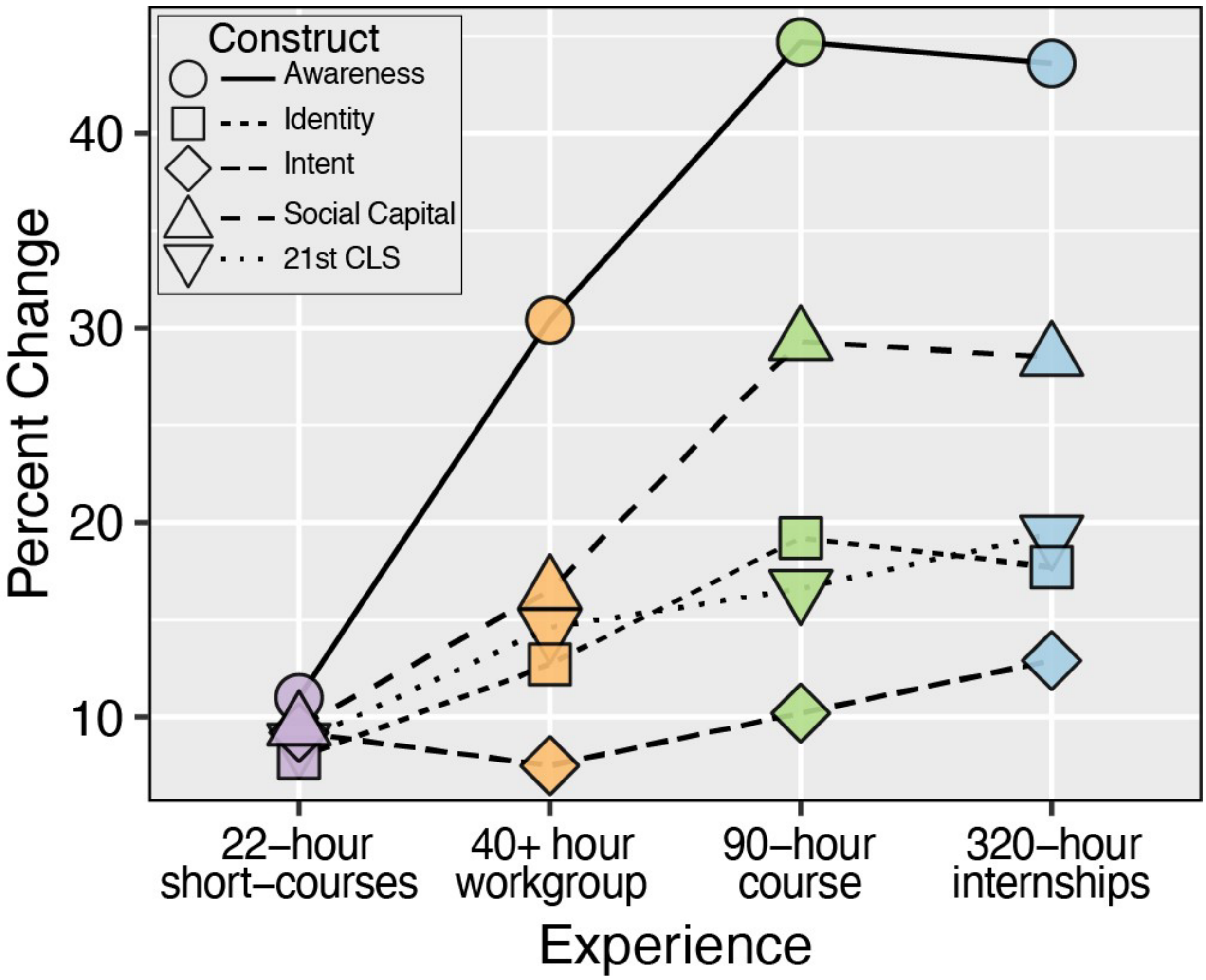

METHODS

Deeper descriptions of terms and experience models

Participants

For the purposes of this study, “alumni” were defined as students who participated in a Systems Education Experience in or before 2019; and “participant data” was collected from students participating in one of four experiences with Systems Education Experience (SEE) in 2019, 2020, or 2021. Due to the advanced

content of the experiences and age requirements for students to work in our laboratory facilities, only rising juniors and seniors were accepted into these immersive experiences.

STEM Experience Model Descriptions

320-hour internships

Summer internship experiences were reserved for rising seniors as this was a full-time (up to 40-hours a week for 8 weeks) paid experience that required a high level of independence and maturity (https://see.isbscience.org/resources/for-students/high-school-intern-program/). The number of students offered an internship was contingent on the number of mentors and the amount of lab/desk space available each year. Internships are typically provided in-person at Institute for Systems Biology (ISB). However, due to in-person restrictions from the COVID-19 pandemic, the 2020 and & 2021 internships were offered remotely. Student interns (in-person and remote) worked with many specialists who directly involved the students in a variety of systems biology research projects that allowed them to gain valuable STEM experience in fields like microbiology, engineering, and computational science. If applicable, student interns worked in partnership with teachers and the specialists to translate their systems research into open-access curriculum modules to be used in schools, formal courses and informal STEM programs (https://see.isbscience.org/about/overview/). Due to the unique nature of this curriculum building and research-based laboratory experience, students engaged in creative problem solving as well as both independent and group learning. In addition to the scientific and educational components of their 8-week internship, students also met with a variety of the SEE staff to explore many types of professions and career paths. To showcase their work and to document their experience, students learned from a skilled web designer to develop their own web pages which are hosted on the SEE website (Systems Education Experiences, 2023b).

90-hour course

In 2019, an in-person 90-hour “STEAM Towards a Healthier World” course was developed and launched over 3 weeks for 18 rising juniors and seniors. The course provided students with the opportunity to explore the relationship between the environment and health and gain understanding of systems approaches in health and medicine. Lessons were led by certified teachers and STEM professionals. A collaboration with the Seattle Science Foundation’s “Anatomy Bootcamp” allowed students to work with neuro and spinal surgeons from around the country to explore the human body through dissections of real human cadaveric specimens (https://see.isbscience.org/steam2019/). At ISB, students engaged in authentic lab-based experiences, participated in discussions, listened to guest speakers on a variety of health-related topics, and completed collaborative projects. At the culmination of the course, students showcased their learning with art projects they developed to teach others about one of the topics that was particularly interesting to them. The projects were shared with the ISB community (MDs, PhDs, MS, etc.), family, and friends at a final showcase event held at ISB, and are featured on the course website (https://see.isbscience.org/steam2019/). Due to the hands-on nature of this course, we were not able to offer this opportunity in 2020 or 2021 due to COVID-19 restrictions.

40+ hour workgroup

To meet the needs of students looking to engage in STEM during the start of the COVID-19 pandemic, in 2020 a virtual Computational Modeling Workgroup was developed and launched with 42 rising seniors and was completed by 41 students (Ludwig, et al, 2020). Students participated in 40 hours of synchronous sessions, and completed an estimated 1 to 45 hours asynchronously based on their interest. Students worked with 3 educators and 15 STEM professionals to learn how computational modeling is used to understand and solve a variety of systems problems. Students also learned about the vast career pathways and jobs available for people with interest and skills in data analytics, computer science and computational biology. While learning, students also designed and completed projects with the online guidance of scientists, programmers, and educators within or connected to ISB. The projects were shared with the ISB community,

family, and friends at a final showcase event held online via Zoom, and are featured on the workgroup website (Ludwig, et al., 2020).

22-hour short-courses

As the number of program applicants continued to increase, so did the exploration of novel ways to meet more students' needs. In 2021, a 2-tiered "Systems Thinkers in STEM Ambassador" (STiSA) remote program was created and launched for rising juniors, seniors, and a small number of students who had just graduated high school (https://see.isbscience.org/stisa-2023-2021/). The 2-tiered online program consisted of a 2-hour workshop (Tier 1) followed by a 20-hour short-course (Tier 2). Every student who completed an application in 2021 and those who completed an application in 2020 but were unable to participate in a program at that time, were invited to participate in the 2021 STiSA program. Of the 686 eligible students, 288 students chose to participate in Tier 1. Of the students who completed Tier 1, 180 chose to continue their participation in Tier 2 for a full 22-hour experience, and 11 chose to continue their learning by participating in a for-credit course offered by SEE staff through a local skills center, Washington Network for Innovative Careers. Due to the unique aspects of the skills center courses, the data and analysis provided for the 22-hour short courses includes the 180 students, but does not include the 11 students who participated in the skills center for-credit course.

Tier 1 workshops were offered 2-3 times monthly from February through April, 2021, for a total of 7, 2-hour Zoom workshops. Tier 1 workshop content aligned with the learning objectives of SEE's career-connected curriculum module "Systems Are Everywhere" (https://see.isbscience.org/modules/systems-are-everywhere/). Activities supported students in learning about systems, systems modeling, and developing systems thinking skills. Tier 2 short-courses were developed as modified 20-hour versions of the SEE modules "Bioengineering a Sustainable World" (BSW - https://see.isbscience.org/modules/bioengineering-a-sustainable-world/), "Gaining Insight through Systems Thinking and Computational Modeling" (CM - https://www.gaininginsight.org/), and "Systems

Medicine" (SM - https://see.isbscience.org/modules/systems-medicine/). These 3 modules were chosen out of SEE's existing 12 modules based on student interest and due to their ability to be delivered online. Based on student interest and feedback, each of the 20-hour short-courses were offered twice and at various days and times for a total of 6 student cohorts (BSW1, BSW2, CM1, CM2, SM1, SM2). Effort was made to assign students to their first choice, if that was not possible, they were invited to their second choice; each student was assigned to a single course.

RESULTS

More on Participants:

The majority of respondents (65.5%) to the Alumni Survey reported having completed their ISB experience between 2015-2019, while the remainder reported having completed their experience between 2010 and 2014 (25.5%) or between 2005 and 2009 (9%). We report this because we recognize that the longer a person is away from an experience the less reliable their retrospective pre-post survey answers may be. We also acknowledge that the majority of interns between 2005 and 2009 remain connected with program staff, so while their initial experience was over 10 years ago, their interactions have likely occurred more recently.

More on Culminating Projects with Student Quotes:

Student projects included (but were not limited to) creating posters describing the results of their research projects (320-hour internships), using art to share their learning with a target audience (90-hour course), applying their computational skills to create a modeling project for others to learn from (40+ hour work group), creating a systems model of factors that impact health, creating a profile describing how they have become a Systems Thinker in STEM, or creating a presentation of a STEM career that uses computational modeling (22-hour short-courses). Students presented their projects to their research group / class; additionally, students participating in the 320-hour, 90-hour and 40-hour experiences shared their projects

at community events, on the SEE website, and via social media. A student who completed the 40+ hour work group experience shared, “Creating my own project…was the most valuable experience and the one that I learned the most from. It was a lot more difficult than expected and, eventually, we did end up pulling together a project which I am very proud of. This gave me a lot of confidence in pursuing a career in STEM if I wanted to!”. Students who completed a 22-hour short course shared the career presentations were “extremely helpful in spotlighting a few careers and the education required for each”, and “helped me learn more about possible stem careers and become more interested in them.”

Projects were often done collaboratively, or if done individually included peer review and feedback. One student acknowledged, “I usually work better alone but I'm glad I asked for a partner on my project, because I think we work better as a team, and the overall project came out so much better than it would have if I did it alone. Not only do they help with the work, but they provide support and additional confidence in the work we're bringing about. I'm also proud to have them as a good friend of mine now!”. Another shared, “Working on our individual projects helped us learn about planning and taking risks. We also collaborated with others and shared ideas about tools and data.”

More Student Alumni Quotes gathered in the Surveys as an extension of Table 4 and Figure 2

These additional Alumni quotes were gathered via the study survey, but were not included the manuscript. The quotes highlighted in grey align with multiple components of the STEM pathways framework. As a reminder, the main STEM pathway components are: 1) make informed decisions about their **course of study**; 2) understand potential STEM **career pathways**; 3) have a clear understanding of STEM **content and practices**; 4) understand potential **barriers** in STEM and how to address them; 5) become more aware of **connections** between STEM and societal issues and concerns.

Career pathways

“Having informal interviews with ISB folks helped me learn about careers in STEM and what educational paths might suit me. The hands-on process of working in the lab helped me understand STEM content and practices.” (2018 Intern)

"I felt as though I got a great introduction to the many different career paths in STEM with the various interviews of members of ISB. It was also very informative and fun to be experience what a full-time job would look like in biology research." (2010 Intern)

"I gained hands on experiences, further expanding my knowledge on STEM content and practices. I was able to learn about the typical type of career in this field and learned even more after interviewing other scientists."(2019 Intern)

"Mentors at ISB really encouraged me to think about interdisciplinary collaborations, since my area of interest was tangential to the biological sciences." (2012 Intern)

Content & practices

"ISB gave me invaluable perspective on what it is to work in a research lab." (2017 Intern)

"ISB was very real-world and continues to inform my thinking about what it means to do research, both as an individual and as an institution." (2010 Intern)

"Having informal interviews with ISB folks helped me learn about careers in STEM and what educational paths might suit me. The hands-on process of working in the lab helped me understand STEM content and practices." (2018 Intern)

"I felt as though I got a great introduction to the many different career paths in STEM with the various interviews of members of ISB. It was also very informative and fun to be experience what a full-time job would look like in biology research." (2010 Intern)

"I gained hands on experiences, further expanding my knowledge on STEM content and practices. I was able to learn about the typical type of career in this field and learned even more after interviewing other scientists."(2019 Intern)

The hands-on process of working in the lab helped me understand STEM content and practices." (*2018 Intern)*

"I had exposure to a world I didnt know existed before the internship." (2016 Intern)

"[I] learned skills that prepared me really well to apply for other research positions in undergrad." (2013 Intern)

"ISB provided an amazing hands-on experience in STEM that reaffirmed by interest in the field. It also gave me valuable experience working in a lab is like which again, reaffirmed by desire to study STEM and helped me obtain other successful research internships." (2015 Intern)

"I truly think the real-world knowledge of what it really looks like to be working in STEM was one of the most valuable takeaways from my internship." (2018 Intern)

General

"My experience at ISB gave me a confidence boost during my time in high school. The encouragement and support I received from my mentors and the other interns was mind blowing." (2016 Intern)

“So the things I learned and saw at ISB are very clearly still with me and shaping how I see the world!” (2010 Intern)

“Skills that I learned at ISB carried over and helped me to be successful and independent at a different internship! I wouldn’t trade my time at ISB for anything, it’s helped me be a lot more knowledgeable and confident in what career I want to pursue.” (2017 Intern)

“I feel that I gained more than real-world experience, but true independence, problem-solving skills, perspective, and awareness. I also want to thank everyone at ISB for being so kind and willing to talk to me. I felt truly respected, as an equal and adult, and it meant a lot to me.” (2018 Intern)

“I have been lucky to attend college with some of my ISB peers. We have taken STEM classes together and even been study buddies for a few courses.” - 2017 Intern
